# Larval Connectivity and the International Management of Fisheries

**DOI:** 10.1371/journal.pone.0064970

**Published:** 2013-06-07

**Authors:** Andrew S. Kough, Claire B. Paris, Mark J. Butler

**Affiliations:** 1 Rosenstiel School of Marine and Atmospheric Sciences, University of Miami, Miami, Florida, United States of America; 2 Department of Biological Sciences, Old Dominion University, Norfolk, Virginia, United States of America; Institute of Marine Research, Norway

## Abstract

Predicting the oceanic dispersal of planktonic larvae that connect scattered marine animal populations is difficult, yet crucial for management of species whose movements transcend international boundaries. Using multi-scale biophysical modeling techniques coupled with empirical estimates of larval behavior and gamete production, we predict and empirically verify spatio-temporal patterns of larval supply and describe the Caribbean-wide pattern of larval connectivity for the Caribbean spiny lobster (Panulirus argus), an iconic coral reef species whose commercial value approaches $1 billion USD annually. Our results provide long sought information needed for international cooperation in the management of marine resources by identifying lobster larval connectivity and dispersal pathways throughout the Caribbean. Moreover, we outline how large-scale fishery management could explicitly recognize metapopulation structure by considering larval transport dynamics and pelagic larval sanctuaries.

## Introduction

The lifecycle of most marine animals includes a dispersive planktonic larval stage lasting hours to months that connects scattered populations. Therefore, knowledge of larval connectivity is crucial for understanding population dynamics and sustainably managing marine taxa whose biogeographic distributions rarely coincide with political boundaries. Recent studies of larval connectivity employing natural or artificial tags [1]–[3], biophysical modeling [4]–[6], tracking of larval patches [7], and genetic analysis [8]–[10] have revealed surprising levels of population self-recruitment, eclipsing the long-held paradigm that marine populations are largely “open” and dependent upon an exogenous supply of larvae [11]. As compelling as these findings are, the ability to predict the actual dispersal of larvae from spawning grounds to nurseries remains a rare exception. Here, we describe how an empirically parameterized biophysical model provides estimates of larval supply and may be used to pinpoint larval origins, destinations, and pathways for one of the Caribbean's most valuable marine species - the spiny lobster, *Panulirus argus*.

The Caribbean spiny lobster is a ubiquitous inhabitant of coral reefs and shallow tropical seas in the tropical West Atlantic. Commercial fishermen and recreational divers in over 30 Caribbean nations harvest lobsters, a resource valued at nearly $1 billion USD annually [12]. Like most marine animals, *P. argus* has a complex life cycle: adults inhabit coral reefs where they spawn, their planktonic larvae (phyllosoma) mature in the open sea and engage in diurnal and ontogenetic vertical migration during dispersal before returning to coastal nurseries in shallow, vegetated habitats [13]. Given the long pelagic larval duration (PLD) of this species (5–9 months) [14], larvae potentially disperse among lobster populations throughout the Caribbean [15]. Genetic analyses support the hypothesis of a single “pan-Caribbean” lobster metapopulation [16]–[18], indistinguishable within the Caribbean but distinct from a closely related species off the coast of Brazil [19].

Frequent and widespread dispersal of larvae can mask genetically distinct subpopulations, whereas demographic connectivity - the frequent (i.e., weeks to years) exchange of individuals within a metapopulation - is a fundamental ecological process relevant to the management of marine fisheries and protected areas [20]. Studies of demographic connectivity have largely focused on taxa with short PLDs (e.g., bivalves and reef fish) and though valuable scientific contributions, they likely bias our understanding of connectivity at the larger spatial scales most important for marine resource management [21]. Demographic connectivity among distant (>1000 km) populations is virtually undetectable given current tagging methods and genetic techniques [22], [23]. For this less tractable circumstance, biophysical modeling is a fast and affordable tool that is unhindered by the PLD of target species; moreover it permits the evaluation of hypothetical management strategies on larval connectivity within marine metapopulations [24].

**Figure 1:**
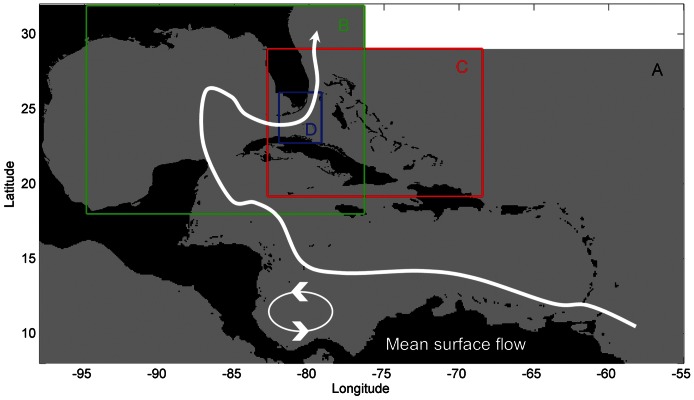
The hierarchy of nested circulation models used in the study and the conceptual mean Caribbean flow. The ocean circulation models used in reverse order of priority for use by the Lagrangian tracking module with their horizontal resolution and vertical depth bins in meters. A) HYCOM Global 1/12 degree: 0, 10, 20, 30, 50, 75, 100; B) GOM-HYCOM 1/25 degree: 0, 5, 10, 15, 20, 25, 30, 40, 50, 60, 70, 80, 90, 100; C) Bahamas ROMS 1/24 degree: 0, 2, 4, 8, 10, 20, 30, 40, 50, 55, 60, 80, 100; D) FLK-HYCOM 1/100 degree: 0, 5, 10, 30, 50, 75, 100. Mean surface flow after Fratantoni [76].

To identify the origins, destinations, and dispersal corridors of spiny lobster larvae within and among Caribbean nations, we used an open source, multi-scale coupled biophysical larval transport model [25] built from an earlier configuration of a Lagrangian individual-based model [26]. The model has four components: 1) a GIS-based benthic module representing habitat for lobster spawning and recruitment, 2) a physical oceanographic module (Figure 1) containing daily 3-D current velocities from an array of hydrodynamic models, 3) a larval biology module depicting larval life history characteristics, and 4) a Lagrangian stochastic module that tracks the trajectory of individual larvae. We parameterized the model with data on spatio-temporal patterns of spiny lobster spawning and planktonic larval behavior, and then verified the model by comparing simulation results with empirical data on the spatio-temporal patterns of larval supply at four sites in the Caribbean (see Methods). Compared to other larval dispersal models created for spiny lobsters [27]–[33], our model uses the highest resolution, three-dimensional oceanographic circulation models and also larval behavior, both of which affect dispersal trajectories [34]. Our objectives were to employ this modeling system to investigate: (a) the demographic connectivity of spiny lobster larvae among Caribbean nations, (b) the international patterns of larval imports and exports, and (c) the relevancy of connectivity in designing Caribbean-wide networks of marine protected areas (MPAs). An unanticipated phenomenon also emerged from our modeling results: the predicted existence of pelagic larval nursery areas.

## Results

### Model Verification

**Figure 2:**
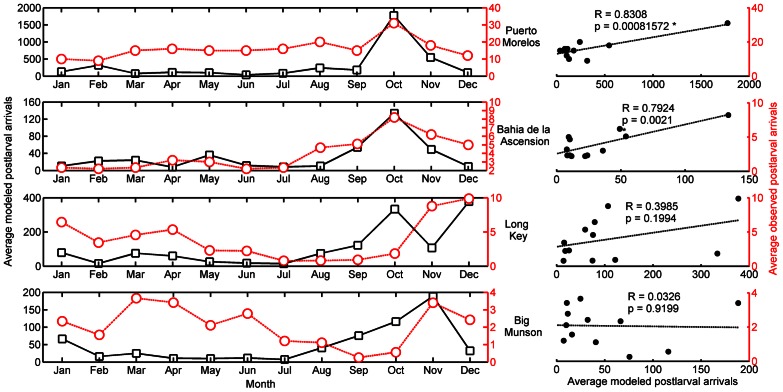
The seasonal pattern of observed postlarval arrival compared to model predictions. A comparison of the actual coastal arrival of *P. argus* postlarvae (red) as compared to model predictions (black) over four years at four different locations (Mexico: Bahia de Ascension, Puerto Morales; Florida: Long Key, Big Munson). The Florida observations [35] are of average postlarval arrivals per collector from 2004–2008. The Mexican observations are averages from Briones-Fourzan [29]. The correlation between the modeled and the observed arrivals was significant (p<0.05) for Bahia de Ascension and Puerto Morales. The model also predicted the appropriate peak month of settlement in three locations, suggesting that the model can capture the temporal pattern of arriving larvae.

Two independent sets of empirical data on postlarval lobster settlement that were not used in the parameterization of our model [29], [35] were subsequently used to evaluate the final coupled system’s performance. The model was compared against the monthly patterns of *P. argus* postlarval arrival at two sites in both Mexico and Florida, corresponding to four separate habitat polygons (sites) in the model (Figure 2). The simulated pattern of monthly arrival of postlarval lobsters was significantly correlated (p<0.05) with observed postlarval recruitment at two of the four sites and captured the peak in seasonal recruitment at all four sites (Figure 2). The model shows the fall peak in postlarval arrival in the Florida Keys, but does not show the spring peak (Long Key and Big Munson; Figure 2).

### Connectivity Matrices

**Figure 3:**
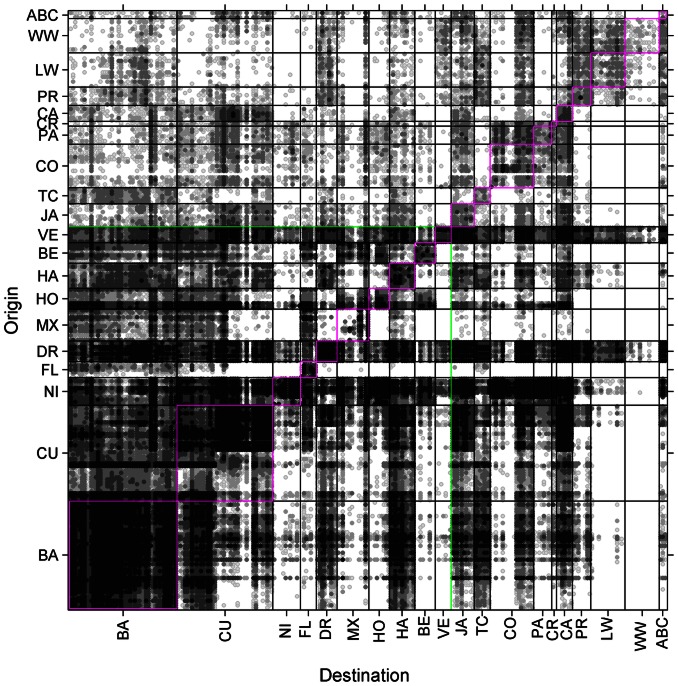
Connectivity matrix of spiny lobster (*P. argus*) larva. A simple matrix showing the number of larva migrating from place to place in a coupled biophysical model. The origin of each larval connection is from the left (rows) and the destination of the larvae is at the bottom (column). Domestic connectivity (recruits that settled into their origin nation) follows the diagonal. The strength of connections among sites is a percentage of the total larval exchanged, and the grey shades represent five quantiles. The top 10 lobster fishery nations are separated by the green box. The results are from four years of Caribbean-wide lobster larval dispersal simulations among 261 habitat sites distributed into 39 countries whose abbreviations are: BA = Bahamas; CU = Cuba; NI = Nicaragua; FL = Florida; DR = Dominican Republic; MX = Mexico; HO = Honduras; HA = Haiti; BE = Belize; VE = Venezuela; JA = Jamaica; TC = Turks and Caicos; CO = Columbia; PA = Panama; CR = Costa Rica; CA = Cayman Islands; PR = Puerto Rico; LW = Leeward Islands (10 countries); WW = Windward Islands (9 countries); ABC = Aruba, Bonaire, and Curacao.

**Figure 4:**
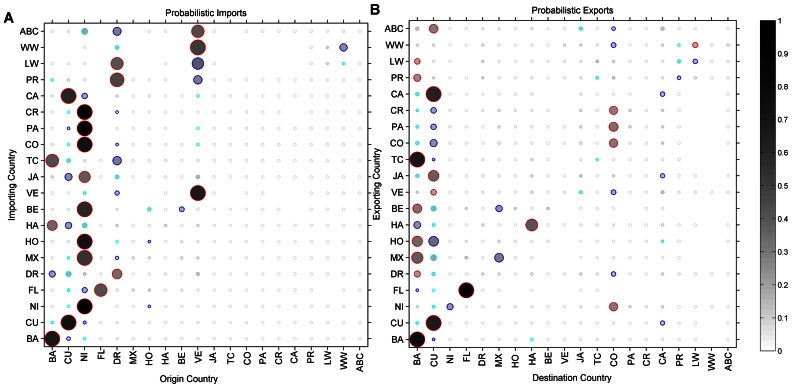
Probabilistic imports (A) and exports (B) of spiny lobster (*P. argus*) larva grouped by political boundaries. The probability for each instance is computed as: 
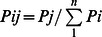
 where *i* = the country importing (A) or exporting (B), *j* = the origin (A) or the destination (B) country, and *n* = all countries. The size and shade of grey of the bubble represent the normalized probability, increasing with size and darkness. The three highest probabilities in each scenario are also colored in red, blue, and cyan, respectively.

Our simulations reveal distinct flows of long-lived spiny lobster larvae among some regions of the Caribbean and pockets of larval retention within others (Figure 3). Probabilistic imports and exports of larvae from each of 261 sites show that the majority of larval exchanges transcend international boundaries when summarized by country (Figure 4). Nonetheless, domestic connectivity (i.e., self-recruitment of lobsters within a country) still dominates larval recruitment in some areas. For example, lobster populations in the Bahamas, Cuba, Nicaragua, and Venezuela are largely self-recruiting, whereas those in the Cayman Islands, Columbia, Honduras, Jamaica, Panama, and Puerto Rico depend largely on larval subsidies from outside their borders.

### Imbalanced International Exchange

**Figure 5:**
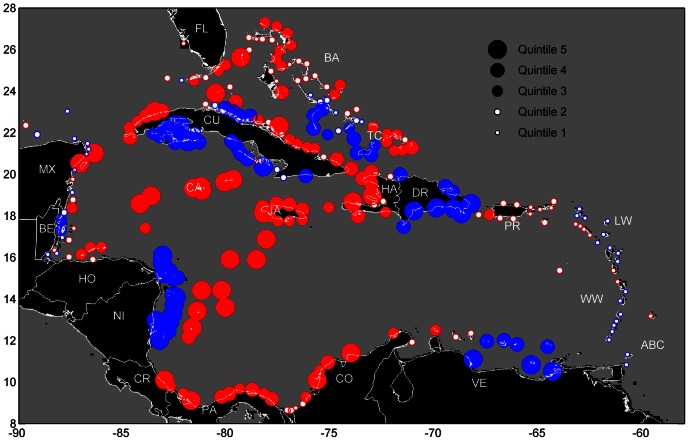
International larval exchange of spiny lobster (*P. argus*) larvae. The difference between larval exports and imports at a site (n = 261), after removing self-recruitment. The size of the circle depicts the relative magnitude of the difference, grouped into 5 quantiles. The direction of the difference is shown as blue for positive (more larval exports) and red for negative (more larval imports). BA = Bahamas; CU = Cuba; NI = Nicaragua; FL = Florida; DR = Dominican Republic; MX = Mexico; HO = Honduras; HA = Haiti; BE = Belize; VE = Venezuela; JA = Jamaica; TC = Turks and Caicos; CO = Columbia; PA = Panama; CR = Costa Rica; CA = Cayman Islands; PR = Puerto Rico; LW = Leeward Islands (10 countries); WW = Windward Islands (9 countries); ABC = Aruba, Bonaire, and Curacao.

Much like international trade, large disparities between larval imports and exports among countries abound in our simulations. We identified imbalances in the international exchange of lobster larvae by removing model predictions of domestic connectivity from the total larval supply and then compared the remaining difference in larval subsidies received and subsidies donated to the pan-Caribbean larval pool (Figure 5). This analysis reveals which countries harbor lobster populations that sustain populations elsewhere. The eastern Bahamas, southern Cuba, Dominican Republic, Nicaragua, and Venezuela export far more lobster larvae than those areas receive from the international community. In contrast, the western Bahamas, Cayman Islands, northern Cuba, Columbia, Florida Keys, Jamaica, and Panama are regions whose lobster populations receive more larvae from outside their boundaries than they donate to the Caribbean larval pool.

### Connectivity and Marine Reserve Networks

Networks of MPAs have been proposed as a solution to ensure that demographic connectivity is maintained among marine animal metapopulations, with a recommendation that on average 20–30% of the coastal seas be set aside as MPAs [36]. We used our model to explore this recommendation specifically for spiny lobster in the Caribbean by designating various model sites as hypothetical MPAs and evaluated different networks of sites as if they were the sole sources of lobster larvae for the Caribbean (Table S1). Five MPA network scenarios were evaluated in simulations in which 40 habitat sites were designated as MPAs and selected in one of five ways: (1) *Random*: 40 sites individually and randomly selected from all those in the Caribbean, (2) *Stratified Random*: two randomly selected sites from each of the 20 countries, (3) *Self-Recruitment:* the top two self-recruiting sites per country, (4) *Long-distance Dispersal*: the top forty sites which successfully export larvae internationally in the Caribbean (5) *Maximum Export*: the top forty sites throughout the Caribbean with export imbalanced exchange (Figure 4). For these simulations the magnitude of larval production from each habitat site was fixed and uniform (unlike the more realistic and variable production used in our first set of simulations), which removed the effect of differences in local population size and focused on the effect of spatial arrangement of MPAs on biophysical connectivity networks. In each of the MPA scenarios, only the larval transport that originated from the 40 selected sites was considered, thus treating the system as a patchwork of MPAs.

The geographical location and connectivity characteristics of sites selected as MPAs altered patterns of spiny lobster larval dispersal and settlement (Table S1). Sites selected at random (scenarios 1 and 2; bootstrapped 1000 times to create averages) produced less successful larval connectivity than sites selected based on their merit as international (scenarios 4 and 5) or domestic (scenario 3) larval exporters. Simulations focusing on preserving domestic connectivity caused a near universal increase in larval recruitment across the Caribbean, although smaller than the ideal internationally managed scenario. Thus, by taking into consideration the complex patterns of connectivity for a species, we can add specificity to the general recommendation that a certain proportion of the sea requires protection to sustain marine fishery resources.

### Pelagic Larval Nurseries

**Figure 6:**
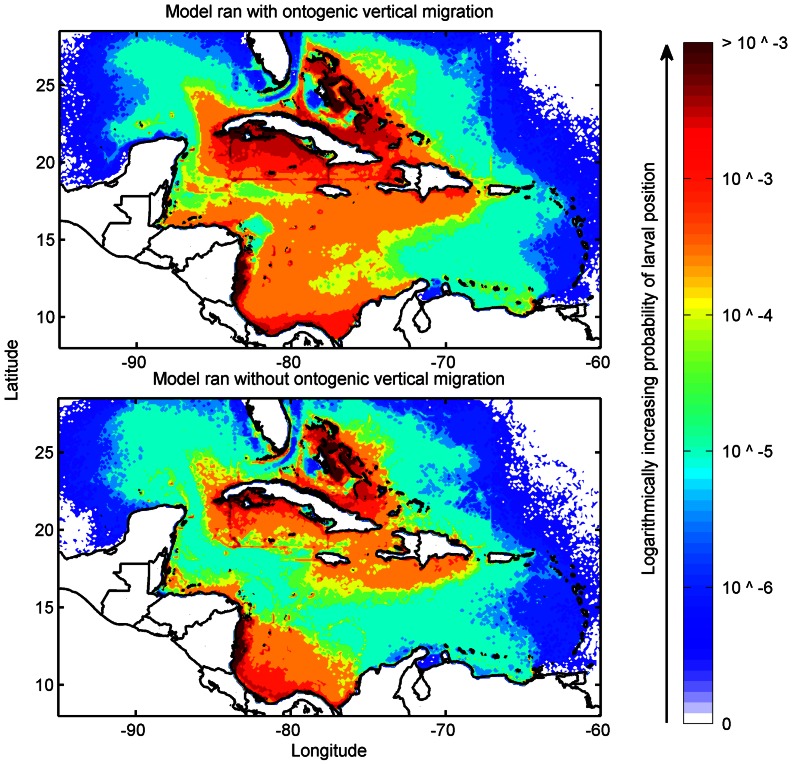
Probabilistic modeled spiny lobster (*P. argus*) larval concentrations. The probability density distributions represent pelagic nursery habitat within the Caribbean Sea for successfully recruiting spiny lobster larvae. The output location was recorded on a ten day frequency and added into a 0.1°×0.1°gridcell. Blue areas were relatively devoid of successfully dispersing larvae; warmer colored regions had more larval trajectories pass through them, increasing logarithmically from blue to red. The most important pelagic nursery zones for larvae are represented in red-orange. The areas of highest mean flow through the Caribbean represent a distinct, inter-linked larval ‘graveyard’. Simulations were conducted with (A; n = 54,186,756 larval locations) and without (B; n = 68,675,786 larval locations) ontogenetic vertical migration.

An unexpected pattern in larval distribution within the open ocean also appeared in our simulations. When we examined the oceanic pathways travelled (i.e., sum of PLD spent in each oceanic locale) by successfully settling larvae in contrast to the paths taken by larvae that are eventually lost from the system, zones emerged that could be described as “pelagic larval nurseries”. That is, regions in the open Caribbean Sea where lobster larvae from around the Caribbean spend much of their planktonic existence before later settling into coastal benthic nurseries. These larval nurseries include relatively large regions offshore of Nicaragua, southern Cuba, and the central Bahamas as well as smaller areas north of Cuba and southeast of Hispaniola (Figure 6). We evaluated the role of larval behavior in creating these pelagic nurseries by conducting an additional simulation without ontogenetic vertical migration (OVM) by larvae, thus simulating passive larval dispersal. The segregation between the regions of concentration was accentuated when larvae drifted passively (Figure 6), indicating that the larval nursery zones were governed primarily by physical oceanographic features, not OVM behavior specific to spiny lobsters. Thus, these pelagic larval nurseries are potentially relevant to the pelagic retention of other Caribbean species, not just spiny lobster.

## Discussion

Managing marine fisheries organisms as if they were constrained within geopolitical boundaries is not working as fisheries worldwide are in decline [37], [38]. For example, in regions where the spiny lobster *P. argus* are most abundant and thus heavily fished, adult stocks have declined by 30% or more over the past two decades despite spirited management [39]–[42]. For many species, an approach to fisheries management that acknowledges dispersal dynamics with estimates of larval connectivity is needed and now possible.

When we used MPAs in our model to “protect” specific locales that tend to export larvae internationally, those simulations yielded the highest successful settlement of lobster larvae throughout the Caribbean. Certain regions contribute disproportionately to the wider Caribbean larval pool, so maintaining the health of spawning stocks in those countries should be an international priority. One strategy for doing so, similar to the trade of “carbon credits” outlined in article 6 of the Kyoto protocol [43], would be to assign each nation “larval credits” based on regional larval export production. Nations that absorb disproportionally more larvae from the international larval pool bear an ethical responsibility and financial incentive to assist in the preservation of spawning stocks in other areas best suited for exporting larvae. Such non-traditional management recommendations are likely to be met with skepticism and their implementation difficult considering the political and economic realities of international agreements and the needs of local communities [44]. Yet scientific evidence suggests that populations of many marine animals persist in an intricate web of metapopulations that are often linked across geopolitical boundaries by larval connectivity and should be managed accordingly.

Just as preserving pathways between habitat fragments is essential for sustaining many terrestrial species [45], intact connectivity corridors for marine organisms may be needed. Our results suggest that larval corridors may exist in the open ocean that regularly concentrate and nurture pelagic larvae during their ontogenetic journey to coastal habitats (Figure 6). In contrast, the prevailing Caribbean current that snakes through the Caribbean Basin appears to be a “graveyard” for larval lobsters. Its high mean flows (Figure 1) presumably entrain and then wash larvae into the North Atlantic where few will survive (Figure 6). This stands in contrast to the view that larvae harness strong currents to successfully disperse long distances [11]. Our simulations with and without larval behavior indicated that the pelagic nursery zones we identified were stable and likely maintained by oceanographic features. Thus, our findings for *P. argus* are likely to be robust despite differences in larval origins, destinations, and avenues of dispersal that invariably differ among taxa with dissimilar dispersive traits [46]. If so, then the existence of pelagic nurseries for larvae has implications beyond lobsters and may constitute consideration as oceanic “essential fish habitat” [47]. Protection of these open ocean larval habitats from potentially deleterious processes (e.g., pollution from oil spills, coastal runoff, and vessel discharges) may be considerations for the long-term sustainability of marine species with dispersive larvae.

Although an adequate flow of larvae among sub-populations is crucial for the sustainability of marine resources, the arrival of larvae at a site does not necessarily equate to successful recruitment. Whereas larval supply and later recruitment are correlated for some species of spiny lobster and in some areas [39]; [48]–[50], unsuitable nursery habitats decouple the relationship between larval supply and juvenile recruitment in others [51], [52]. The transition from pelagic larva to benthic juvenile and on to adulthood is dependent on a variety of post-settlement processes [53], many of which are site-specific and not accounted for in models like ours that assume homogeneous and static habitat quality. Other studies indicate that phenotype-environment mismatches between settlers from one region into another can also contribute to post-settlement mortality and be a barrier to population connectivity [54]. Thus, the integration of biophysical larval dispersal models with spatially-explicit and dynamic depictions of benthic habitat conditions that drive benthic population dynamics [55], [56] are a logical next step in the development of predictive large-scale metapopulation models.

Advances in computing, genetics, and oceanographic remote sensing are yielding tools useful in addressing questions about the connectivity of marine metapopulations that were unfathomable only a decade ago. The dispersal of long-lived larvae is a complex function of temporally unstable hydrodynamics and ontogenetically variable larval behavior. Therefore, models that do not capture these essential system traits or whose results are not verified with empirical data will be misleading. Management of marine resources should benefit from new tools such as biophysical modeling that quantify larval connectivity and thus can be used to help guide policy. For example, the establishment of MPA networks in ecologically relevant areas that maximize larval production and connectivity among disparate populations will maximize population viability in both self-recruiting regions as well as regions dependent upon larvae from elsewhere. Our findings with respect to spiny lobster connectivity in the Caribbean suggest that international management agreements that recognize the existence of marine metapopulations, focus on rebuilding and sustaining adequate spawning stocks [57], and protect sensitive coastal and pelagic nurseries [42] represent a scientifically sound policy for sustainable management of many marine resources.

## Methods

Focusing on the Caribbean's most valuable fishery resource as a model system, we investigated larval dispersal through the use of an open-source coupled biophysical larval transport model, specifically parameterized using empirical data collected for *P. argus* (Table S2). Our model adheres to the recommended practices for Lagrangian biophysical modeling laid forth in North *et al* [58], while also incorporating empirical data for biological parameterization. Empirical estimates of spawning population (this study), laboratory and field observations of larval vertical migration in the water column [59], and postlarval sensory behavior [60] were used to parameterize the early life history traits of *P. argus*. Each of four submodules was specifically parameterized for spiny lobster larvae.

### The Lagrangian Stochastic Module

The Lagrangian stochastic module drives the coupled biophysical Connectivity Modeling System (CMS). It uses a 4th order Runga-Kutta integration scheme [25] in both time and space to improve the accuracy of simulated larval trajectories as is best practice [61]. For each particle, the next position along the trajectory was calculated during each integration time-step of 2700 seconds, comparable to a previous experiment using spiny lobster that used a time-step of 4500 seconds [59]. The trajectories resulting from the modeled time-step and turbulence are smooth and relatively free of artifacts. Submesoscale turbulent movement was accounted for with stochastic turbulent diffusion during each time-step [25], calculated by multiplying a random number between 0 and 1 by the square root of twice the diffusivity coefficient (0.1 m^2^/s) divided by the time-step. We ran simulations starting daily from January 1, 2004 until December 31, 2007, tracking larval flow for over 4 years. Details on the coupled biophysical algorithms and modeling approach can be found in Paris *et al.* [25].

### The Physical Oceanographic Module

The physical oceanographic module contains the various oceanographic models that provide the currents with which to move larvae. These currents vary as depth changes from the surface down to 100 m, which is the likely maximum depth utilized by lobster phyllosoma [59]. A hierarchy of ocean circulation models are nested offline in the physical oceanographic module, allowing a Caribbean-wide simulation scale (–100:−55 degrees longitude West and 8∶32 degrees latitude North) while not compromising resolution in areas with advanced local circulation models (Figure 1). Four different ocean circulation models were nested together for this study: 1/12 degree HYCOM+NCODA Global Hindcast Analysis [62] provided the base, followed by the higher resolution HYCOM+NCODA Gulf of Mexico 1/25° Analysis (GOMl0.04) [63], a 1/24^th^ degree ROMS model of the Bahamas [64], and the fine scale 900 meter resolution FLKeys-HYCOM [65].

### The GIS-based Benthic Module

The GIS-based benthic module determines where larvae can settle and the location, quantity, and timing of larval release. It is directly coupled to the particle tracking module and is accessed during each integration time step. It consists of 261 habitat sites (polygons - vector GIS data) that are a combination of settlement habitat and a sensory envelope reflecting the threshold at which lobster postlarvae can detect and move to settlement habitat (Figures S1–S20 in File S1). Further information on polygon theory is in Paris *et al* [6]. The 18km sensory envelope for this study was constructed based on the sensory abilities of spiny lobster postlarvae [60]. Postlarvae are the highly mobile, non-feeding, settlement stage of spiny lobsters and are capable of detecting nursery habitat cues over similarly long distances [66]. Lobster benthic habitats were delineated based on data from the Millennium Reef Project [67]. Larvae were released from the nearest non-land location to the center of each habitat site.

The daily timing and magnitude of lobster spawning and thus larval release from each habitat site was estimated as a function of lobster density, sex ratio, size, and fecundity. First, the relative abundance of adult lobsters within each Caribbean country was estimated from FAO fishery landing statistics and an independent mail survey. Data was gathered from the top 10 lobster fishing nations that make up 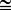
95% of the fishery in the Caribbean. We assumed that the FAO [40] fishery landing statistics are an indicator of relative adult lobster abundance due to the overexploited nature of spiny lobster fisheries. However, these are fishery dependent data with unknown bias (e.g., under reporting of total catch) that may well vary among countries in an unpredictable manner. If so, then the magnitude of larval release in our model and our conclusions would be similarly biased. Unfortunately, there are no other data sources available and these data for *P. argus*, the most prized fishery in the Caribbean, are among the very best for any Caribbean species. The data we obtained on the relative abundance of lobsters among nations was then supplemented by a mail survey distributed to lobster scientists and fishery managers around the Caribbean with intimate knowledge of their local jurisdiction [68]. These data sources provided fine-scale resolution of the timing of spawning, the sex ratio, and the size-structure of adult male and female lobsters, which we used to determine fecundity [69] per habitat site.

**Figure 7:**
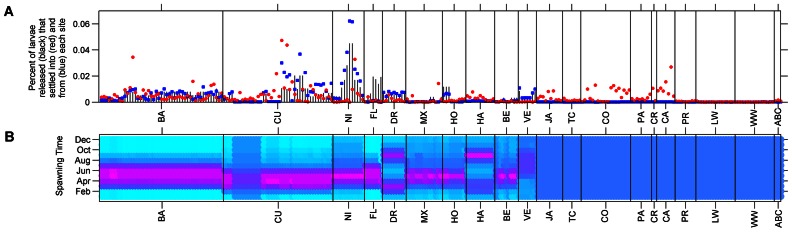
Simulation larval release, settlement, and seasonality. The details of the timing and magnitude of the simulated releases and the larvae received at each habitat site (n = 261). The annual release (black lines), the larvae successfully received (red circles), and larvae donated (blue squares) at each habitat site as a percentage of the total (A). The annual timing of spawning at each site (B). The monthly effort increases from cyan to a peak of spawning occurring in red for locations with dynamic reproductive seasons. A uniform spawning pattern was used in locations that did not have empirical data on spawning time.

**Figure 8:**
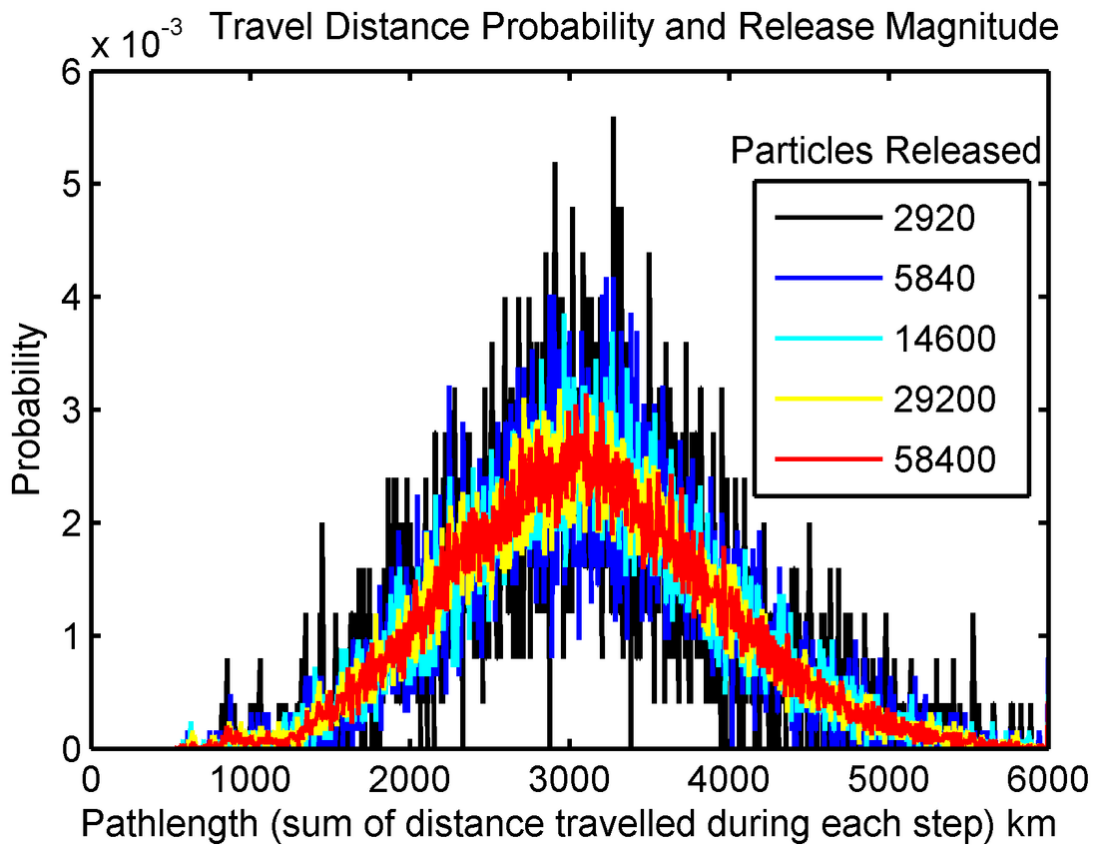
Larval release magnitude and movement pathlength. The probability of dispersal distances for larval releases from a central Caribbean release location (-68°W,14°N). The X-axis is the pathlength (sum of distances moved during each time-step) traveled by each larva binned into 5km increments, and the Y-axis is the probability. The number of larvae released (over 4 years of daily releases) increases in color from black to red and yellow. The smoother curves in red and yellow reflect the stochastic saturation, and suggest the proper number of larvae needed to release daily to probabilistically describe potential lobster larvae dispersal. These values reflect the number of larvae from a single site with no mortality, and had to be multiplied to account for each site and for mortality.

Using these data, we scaled the larval production per habitat site per day proportional to the total annual egg production in the Caribbean (Figure 7A). These estimates of total *P. argus* egg production per year in the Caribbean were then divided into monthly patterns of spawning for each region based on the FAO and survey data (Figure 7B). The total spawned per month and site was further divided into each day because *P. argus* does not spawn synchronously. Finally, we scaled these empirical estimates so as to restrict the annual release of particles in the model to approximately 40,000,000; of which 38,000,000 were distributed to the 10 countries representing 95% the fishery and the remaining 2,000,000 particles distributed equally throughout the rest of the habitat sites with less accurately known lobster population structure. The annual value of 40,000,000 particles was found *a priori* to saturate movement paths in the model, after accounting for mortality (Figure 8). The end result is a daily release of larvae that varied in magnitude proportional to the total fishery, constructed with the local size, population, and spawning patterns when known for each of the 261 habitat sites.

A modified pattern was used to test for idealized MPA placement, which assumed that each habitat site could hold the same climax population size and have the same reproductive potential. This had timing structured as in the original release, but allocated an equal number of particles to each site, rather than scaling population size based on survey and fishery data.

### The Larval Biology Module

The larval biology module accounts for the early life history traits of spiny lobster including PLD, larval competency period, and ontogenetic vertical migration. Lobster larvae display distinct patterns of vertical distribution throughout ontogeny, which greatly alters which currents they are exposed to and therefore their dispersal. To reproduce this behavior, CMS assigns larvae probabilistically to different depth bins [25]. In the present simulations, individual larvae may reside during each time-step within one of five depth ranges (0–20 m, 20–40 m, 40–60 m, 60–80 m, and >80 m) with an age-dependent probability. During each time step, the depth bin is assigned randomly from the age-specific distribution [59]. However, larvae are not allowed to travel more than one depth bin per time step. Older larvae (>3 months old) have a higher chance of being deeper than younger larvae. These probabilities were determined through a combination of plankton trawls and laboratory experiments described in another study [59]. The mean PLD of lobster larvae was observed to be (±1 SD) 174±22 d, based on data from laboratory rearing of *P. argus* from egg to postlarval stage [14]. Larvae in the model metamorphose to postlarvae within a competency period (152 to 196 d) and postlarvae are recorded as ‘settled’ if they enter a benthic nursery habitat site (habitat module) within this competency period; if suitable habitat was not encountered within the competency period they ‘die’ and are removed from the simulation.

Mortality is a key parameter in biophysical modeling [70]. There is no evidence that vertebrate plankton mortality rates are similar to that of invertebrate plankton, however there is growing evidence that mortality changes throughout ontogeny for both coral [71] and fish [72]. To impose mortality, we used a half-life function to reflect varying survivorship as a function of larval duration. There are no known mortality rates for *P. argus* phyllosoma, thus we used an estimate for another spiny lobster (*P. cygnus*) used in Feng *et al* [28], based on trawl surveys that had diminishing returns of later stage larva [73], suggesting abundance based mortality of 85–90%. The cumulative mortality imposed on the larva in our model is ≈ *ca.* 90%, including advective mortality resulting from not reaching settlement habitat.

### The Verification of Our Model

The verification of our model lends credence to its results. Whereas the backbone of coupled bio-physical models are ocean circulation models whose physical dynamics have been validated and peer reviewed, the biological predictions of larval dispersal models should also be verified [74] but few are. Our verification of the model predictions is based on correlations between model predictions and empirical observations of recruitment into relatively small ca. ≈50km^2^ habitat patches following the dispersal of larvae over thousands of kilometers during their 5–9 month PLD (Figure 2). There is precedent for using postlarval collector seasonal settlement trends to verify a Lagrangian model [28], and predictable seasonal patterns are vital for fishery management. Correlating the spatial concentration of observed pelagic larval or juvenile patches with modeled outputs has been done in smaller scale studies [5], [7], [33], [75], but is prohibitively costly and difficult to do at a Caribbean-scale which our model is based on.

Sensitivity analyses of some parameters for which empirical data are lacking or based on laboratory studies (e.g., mortality, PLD, age of competency) could potentially improve the accuracy of our model [6]. Incorporating specific biological traits, for example vertical migrations, into a model alters outputs. For example, Briones-Fourzan *et al* [29] used stochastic perturbations of a particle backtracking simulation to investigate potential origins of postlarvae arriving on the Mexican Quintana Roo coast, without having data on ontogenic vertical migrations. In comparison with their findings, our results suggest diminished larval supply to Mexico from the Lesser Antilles Caribbean Islands and the Venezuelan corridor, while increasing the supply of larva from Central America and Hispaniola (Figure S21 in File S1). This was expected since the vertical migratory behavior of the actively moving larvae increases retention [59]. A simulation that we conducted without larval OVM nor adult population structure did not capture the seasonal recruitment pattern evident in the empirical data (Figure S22 in File S1), and is more similar to the connectivity described in Briones-Fourzan *et al* [29], suggesting that additional biological parameterization could further improve model performance.

## Supporting Information

Table S1 **Strategies for selecting marine protected areas.** Five MPA network scenarios were evaluated in simulations in which 40 habitat sites were designated as MPAs and selected in one of five ways: (1) *Random*: 40 sites individually and randomly selected from all those in the Caribbean, (2) *Stratified Random*: two randomly selected sites from each of the 20 countries, (3) *Self-Recruitment:* the top two self-recruiting sites per country, (4) *Long-distance Dispersal*: the top forty sites which successfully export larvae internationally in the Caribbean (5) *Maximum Export*: the top forty sites throughout the Caribbean with export imbalanced exchange [Fig. 4]. The random sites are the averages of 1000 random selections (Matlab rand function). In each case, an equal number of larvae were released so the difference between scenarios is where the larvae were released from.
(DOC)

Table S2 **Parameterization of the Biophysical Model.** The data used to parameterize each module of the model, along with specific references to sources.
(DOC)

File S1 **Contains Figures S1 to S22.: Figure S1 to S20 in File S1. Habitat maps used for the simulation.** For each country the habitat sites are shown. Sites within each country are numbered according to the location on the previous figures (2 and 3) reading from left to right along the X axis. All axes are latitude and longitude. BA = Bahamas; CU = Cuba; NI = Nicaragua; FL = Florida; DR = Dominican Republic; MX = Mexico; HO = Honduras; HA = Haiti; BE = Belize; VE = Venezuela; JA = Jamaica; TC = Turks and Caicos; CO = Columbia; PA = Panama; CR = Costa Rica; CA = Cayman Islands; PR = Puerto Rico; LW = Leeward Islands (10 countries); WW = Windward Islands (9 countries); ABC = Aruba, Bonaire, and Curacao. **Figure S21 in File S1. A comparison of possible larval sources to the Mexican Quintana Roo coast between two Lagrangian individual based models.** The origins of larvae that arrived to habitat on the Quintana Roo coast during April, May, September, and October. Results from Briones-Fourzan are averages between their figures 10 and 11 [29]. Results from our study, using a simulation with passive larvae released equally in magnitude and timing from around the Caribbean (red), and another incorporating vertical migration behavior and larvae released based on reproductive biology (blue). **Figure S22 in File S1. The seasonal pattern of observed postlarval arrival compared to modeled predictions, without considering population structure.** A comparison of the actual coastal arrival of *P. argus* postlarvae (red) as compared to modeled predictions (**black**) over four years at four different locations (Mexico: Bahia de Ascension, Puerto Morales; Florida: Long Key, Big Munson). The Mexican observations are averages from Briones-Fourzan [29]. The Florida FWC observations [35] are of average postlarval arrivals per collector from 2004–2008. The model parameterization ignored seasonal reproductive characteristics and population sizes. There was no significant (p<0.05) correlation between the modeled and the observed arrivals for any site, highlighting the importance of using spatially and temporally explicit biological knowledge of reproduction in population modeling.
(ZIP)
