# Supplementary material for: Larval Connectivity and the International Management of Fisheries
Source: PLoS One. 2013 Jun 7;8(6):e64970. doi: 10.1371/journal.pone.0064970 (PMC3676408; doi:10.1371/journal.pone.0064970)
Supplement: Table S1 — Strategies for selecting marine protected areas. Five MPA network scenarios were evaluated in simulations in which 40 habitat sites were designated as MPAs and selected in one of five ways: (1) Random: 40 sites individually and randomly selected from all those in the Caribbean, (2) Stratified Random: two randomly selected sites from each of the 20 countries, (3) Self-Recruitment: the top two self-recruiting sites per country, (4) Long-distance Dispersal: the top forty sites which successfully export larvae internationally in the Caribbean (5) Maximum Export: the top forty sites throughout the Caribbean with export imbalanced exchange [Fig. 4]. The random sites are the averages of 1000 random selections (Matlab rand function). In each case, an equal number of larvae were released so the difference between scenarios is where the larvae were released from. (DOC) [file pone.0064970.s001.doc]

|  | Random | Stratified Random | Long-distance Dispersal | Maximum Export | Self-Recruitment |
| --- | --- | --- | --- | --- | --- |
| Total Settled | 2188330 | 214646 | 403889 | 289080 | 274940 |
| Average per site | 838 | 822 | 1547 | 1107 | 1053 |
