# Supplementary material for: Larval Connectivity and the International Management of Fisheries
Source: PLoS One. 2013 Jun 7;8(6):e64970. doi: 10.1371/journal.pone.0064970 (PMC3676408; doi:10.1371/journal.pone.0064970)
Supplement: Table S2 — Parameterization of the Biophysical Model. The data used to parameterize each module of the model, along with specific references to sources. (DOC) [file pone.0064970.s002.doc]

| Module | Trait | Source |
| --- | --- | --- |
| Habitat | Benthic Habitat | Andréfouët et al. 2004 [1] |
| Habitat | Polygon Theory | Paris et al. 2005 [2] |
| Habitat | 18km Postlarval Sensory Buffer | Goldstein and Butler 2009 [3] |
| Habitat | Adult General Abundance | FAO 2006 [4] |
| Habitat | Adult General Abundance | CRTR survey: Participants [5] |
| Habitat | Spawning Timing | CRTR survey: Participants [5] |
| Habitat | Size Based Fecundity | Bertelsen and Matthews 2001 [6] |
| Ocean | Base Hydrodynamic Nest/Layer 1 | HYCOM + NCODA Global 1/12° [7] |
| Ocean | Hydrodynamic Nest/Layer 2 | HYCOM + NCODA Gulf of Mexico 1/25° [8] |
| Ocean | Hydrodynamic Nest/Layer 3 | Bahamas ROMS simulation [9] |
| Ocean | Hydrodynamic Nest/Layer 4 | HYCOM-FLKeys [10] |
| Biology | Max Pelagic Larval Duration (196 days) | Goldstein *et al.* 2008 [11] |
| Biology | Competency (152 days) | Goldstein *et al.* 2008 [11] |
| Biology | Mortality (halflife of 80 days) | Maximum possible while still maintaining saturation [Figure 8] |
| Biology | Vertical Migration Behavior | Butler *et al.* 2011 [12] |
| Lagrangian | Biophysical Approach and algorithms | Paris *et al.* 2013 [13] |
| Lagrangian | Time-step (2700 seconds) | Increased resolution from Butler *et al* 2011 [12] |
| Lagrangian | Horizontal Diffusivity (0.1 m^2/s) | Okubo 1971 [14] |
| Lagrangian | Vertical Diffusivity (0.0005 m^2/s) | Ledwell 1993 [15] |
| Lagrangian | Annual particles released (40 million) | [Figure 8] and methods |

References:

1. Andréfouët S, Muller-Karger FE, Robinson JA, Kranenburg CJ, Torres-Pulliza D, et al. (2004) In: 10th ICRS. Global assessment of modern coral reef extent and diversity for regional science and management applications: a view from space. Okinawa, Japan: Japanese Coral Reef Society pp. 1732-1745.

2. Paris CB, Cowen RK, Claro R, Lindeman KC (2005) Larval transport pathways from Cuban spawning aggregations (Snappers; *Lutjanidae*) based on biophysical modeling. Mar Ecol Prog Ser 296: 93-106.

3. Goldstein JS, Butler MJ, IV (2009) Behavioral enhancement of onshore transport by post-larval Caribbean spiny lobster (*Panulirus argus*). Limnol Oceanogr 54: 1669-1678.

4. Food and Agriculture Organization (2006) Fifth regional workshop on the assessment and management of the Caribbean spiny lobster. Available: ftp://ftp.fao.org/docrep/fao/010/a1518b/a1518b00.pdf Accessed 2013 Jan 1.

5. International collaborators who assisted us by completing surveys (2008) or providing information on adult lobster population structure within their countries include: Karl Aiken (Jamaica), James Azueta (Belize), Julio Baisre and Raul Cruz (Cuba), Richard Beaver (Florida), Nelson Ehrhardt (Nicaragua), Alejandro Herrera (Dominican Republic), Lester Gittens (Bahamas), Nilda Jimenez (Puerto Rico), Kathy Lockhart (Turks and Caicos), Alicia Medina (Honduras), Renaldy Navarro (Nicaragua), Hazel Oxenford (Barbados), Paul Phillip (Grenada), Juan Posada (Venezuala), Martha Prada (Columbia), Lionel Reynal (Martinique and Guadeloupe), Maria Romero, Christine Shing (British Virgin Islands), and Eloy Sosa (Mexico).

6. Bertelsen RD, Matthews TR (2001) Fecundity dynamics of female spiny lobster (*Panulirus argus*) in a south Florida fishery and Dry Tortugas National Park lobster sanctuary. Mar Freshwater Res 52: 1559-1565.

7. Dataset from HYCOM Consortium, HYCOM + NCODA Global 1/12°. Available: http://tds.hycom.org/thredds/global_combined/glb_analysis_catalog.html. Accessed: 2013 Apr 16.

8. Dataset from HYCOM Consortium, HYCOM + NCODA Gulf of Mexico 1/25° Analysis (GOMl0.04). Available: http://tds.hycom.org/thredds/GOMl0.04/expt_20.1.html. Accessed: 2013 Apr 16.

9. Cherubin, LM (2013) High-resolution simulation of the circulation in the Bahamas and Turks and Caicos Archipelagos. Progress in Oceanography: In press.

10. Kourafalou VH, Kang H (2012) Florida Current meandering and evolution of cyclonic eddies along the Florida Keys Reef Tract: Are they interconnected? J Geophys Res: doi:10.1029/2011JC007383

11. Goldstein JS, Matsuda H, Takenouchi T, Butler MJ, IV (2008) The complete development of larval Caribbean spiny lobster, *Panulirus argus*, in culture. J Crustacean Biology 28: 306-327.

12. Butler MJ, IV, Paris CB, Goldstein JS, Matsuda H, Cowen RK (2011) Behavior constrains the dispersal of long-lived spiny lobster larvae. Mar Ecol Prog Ser 422: 223-237.

13. Paris CB, Helgers J, Van Sebille E, Srinivasan A (2013) The Connectivity Modeling System: A probabilistic modeling tool for the multi-scale tracking of biotic and abiotic variability in the ocean. Environ Modell Softw. DOI: 10.1016/j.envsoft.2012.12.006

14. Okubo A (1971) Ocean diffusion diagrams. Deep Sea Res 18: 789-802.

15. Ledwell JR, Watson AJ, Law CS (1993) Evidence for slow mixing across the pycnocline from an open-ocean tracer-release experiment. Nature: doi:10.1038/364701a0
