# Supplementary figures and images for: Larval Connectivity and the International Management of Fisheries
Source: PLoS One. 2013 Jun 7;8(6):e64970. doi: 10.1371/journal.pone.0064970 (PMC3676408; doi:10.1371/journal.pone.0064970)

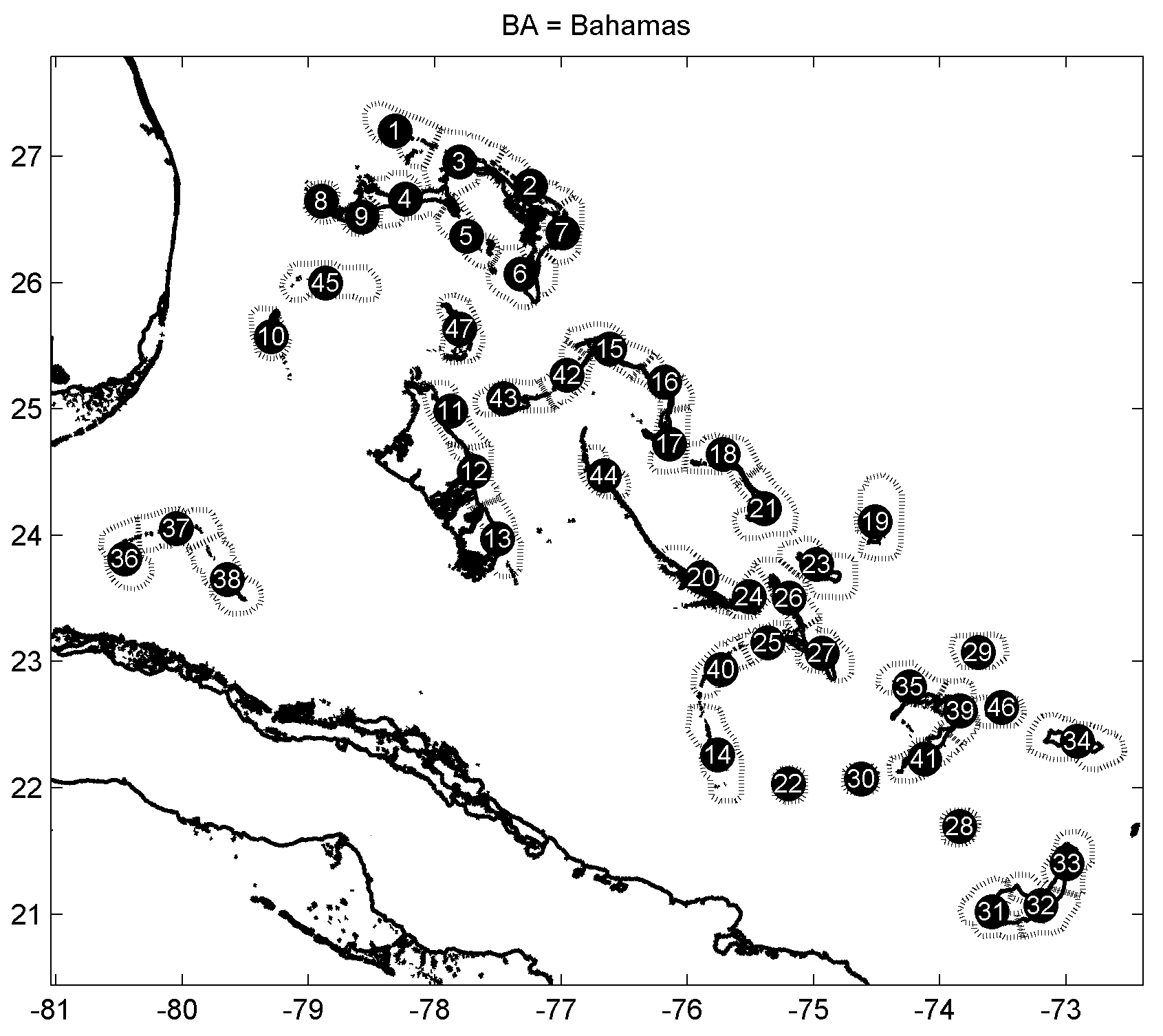

Supplement: File S1 — Contains Figures S1 to S22.: Figure S1 to S20 in File S1. Habitat maps used for the simulation. For each country the habitat sites are shown. Sites within each country are numbered according to the location on the previous figures (2 and 3) reading from left to right along the X axis. All axes are latitude and longitude. BA = Bahamas; CU = Cuba; NI = Nicaragua; FL = Florida; DR = Dominican Republic; MX = Mexico; HO = Honduras; HA = Haiti; BE = Belize; VE = Venezuela; JA = Jamaica; TC = Turks and Caicos; CO = Columbia; PA = Panama; CR = Costa Rica; CA = Cayman Islands; PR = Puerto Rico; LW = Leeward Islands (10 countries); WW = Windward Islands (9 countries); ABC = Aruba, Bonaire, and Curacao. Figure S21 in File S1. A comparison of possible larval sources to the Mexican Quintana Roo coast between two Lagrangian individual based models. The origins of larvae that arrived to habitat on the Quintana Roo coast during April, May, September, and October. Results from Briones-Fourzan are averages between their figures 10 and 11 [29]. Results from our study, using a simulation with passive larvae released equally in magnitude and timing from around the Caribbean (red), and another incorporating vertical migration behavior and larvae released based on reproductive biology (blue). Figure S22 in File S1. The seasonal pattern of observed postlarval arrival compared to modeled predictions, without considering population structure. A comparison of the actual coastal arrival of P. argus postlarvae (red) as compared to modeled predictions (black) over four years at four different locations (Mexico: Bahia de Ascension, Puerto Morales; Florida: Long Key, Big Munson). The Mexican observations are averages from Briones-Fourzan [29]. The Florida FWC observations [35] are of average postlarval arrivals per collector from 2004–2008. The model parameterization ignored seasonal reproductive characteristics and population sizes. There was no significant (p<0.05) correlation between the modele [file pone.0064970.s003.zip › FileS1/S1.tif]

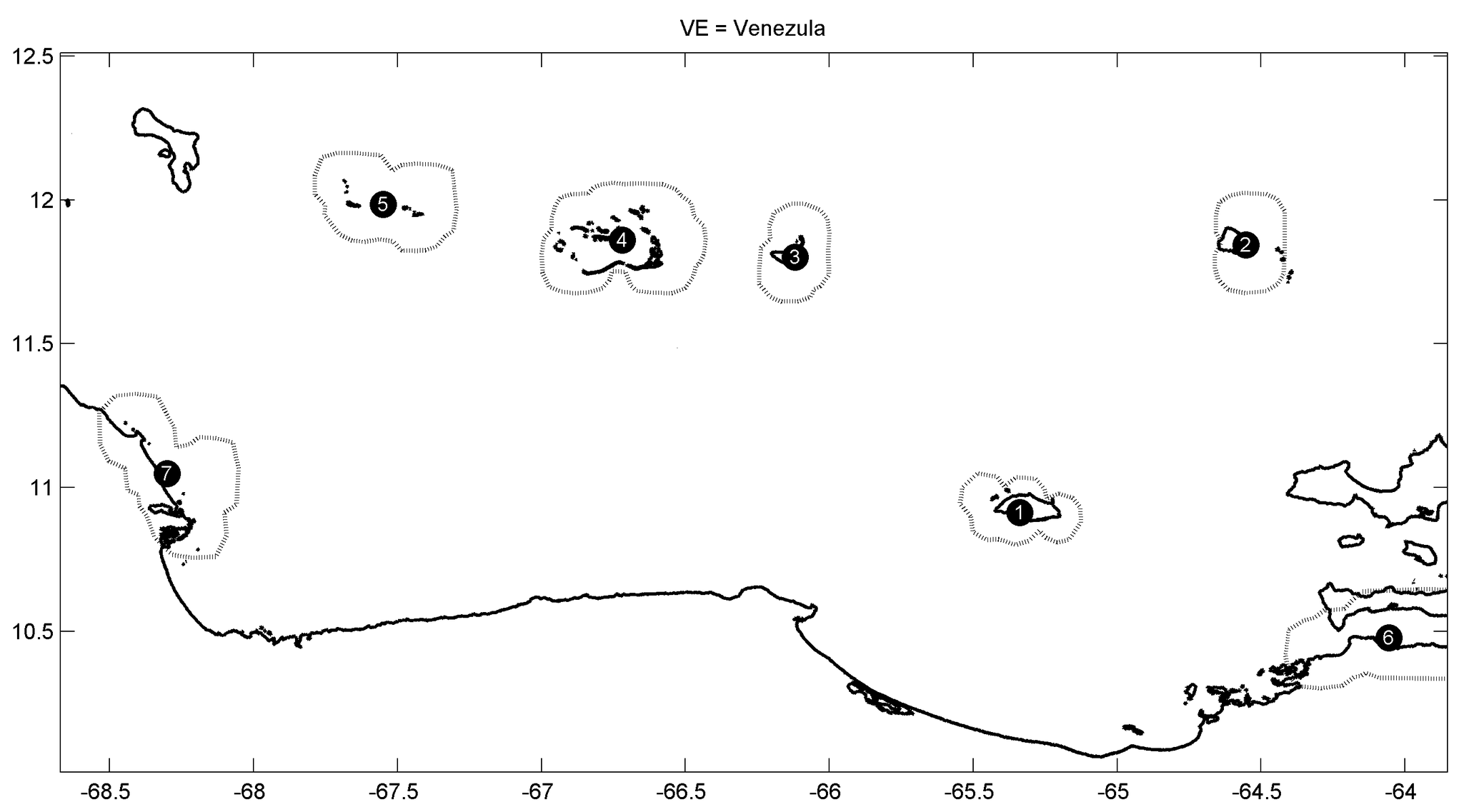

Supplement: File S1 — Contains Figures S1 to S22.: Figure S1 to S20 in File S1. Habitat maps used for the simulation. For each country the habitat sites are shown. Sites within each country are numbered according to the location on the previous figures (2 and 3) reading from left to right along the X axis. All axes are latitude and longitude. BA = Bahamas; CU = Cuba; NI = Nicaragua; FL = Florida; DR = Dominican Republic; MX = Mexico; HO = Honduras; HA = Haiti; BE = Belize; VE = Venezuela; JA = Jamaica; TC = Turks and Caicos; CO = Columbia; PA = Panama; CR = Costa Rica; CA = Cayman Islands; PR = Puerto Rico; LW = Leeward Islands (10 countries); WW = Windward Islands (9 countries); ABC = Aruba, Bonaire, and Curacao. Figure S21 in File S1. A comparison of possible larval sources to the Mexican Quintana Roo coast between two Lagrangian individual based models. The origins of larvae that arrived to habitat on the Quintana Roo coast during April, May, September, and October. Results from Briones-Fourzan are averages between their figures 10 and 11 [29]. Results from our study, using a simulation with passive larvae released equally in magnitude and timing from around the Caribbean (red), and another incorporating vertical migration behavior and larvae released based on reproductive biology (blue). Figure S22 in File S1. The seasonal pattern of observed postlarval arrival compared to modeled predictions, without considering population structure. A comparison of the actual coastal arrival of P. argus postlarvae (red) as compared to modeled predictions (black) over four years at four different locations (Mexico: Bahia de Ascension, Puerto Morales; Florida: Long Key, Big Munson). The Mexican observations are averages from Briones-Fourzan [29]. The Florida FWC observations [35] are of average postlarval arrivals per collector from 2004–2008. The model parameterization ignored seasonal reproductive characteristics and population sizes. There was no significant (p<0.05) correlation between the modele [file pone.0064970.s003.zip › FileS1/S10.tif]

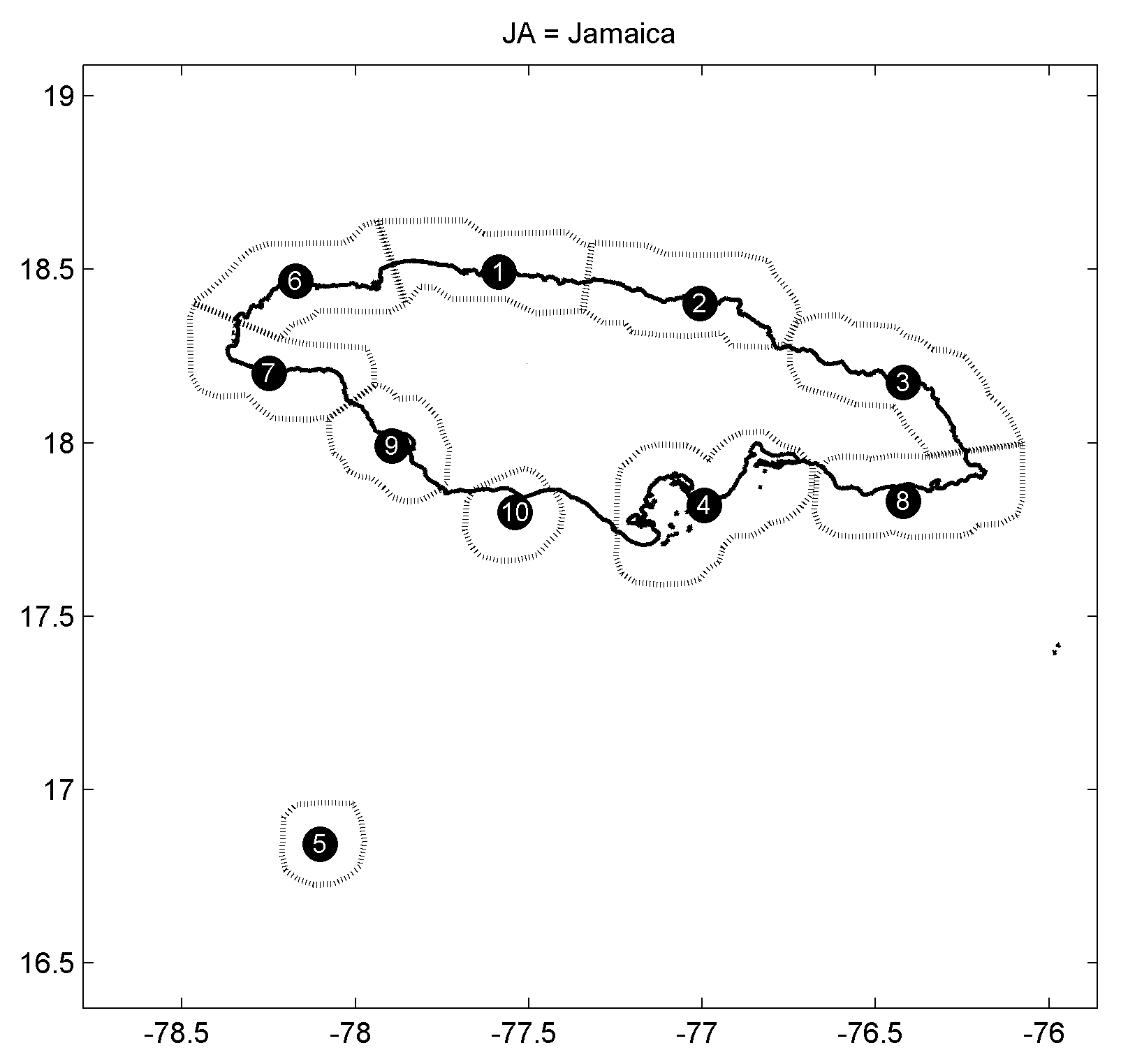

Supplement: File S1 — Contains Figures S1 to S22.: Figure S1 to S20 in File S1. Habitat maps used for the simulation. For each country the habitat sites are shown. Sites within each country are numbered according to the location on the previous figures (2 and 3) reading from left to right along the X axis. All axes are latitude and longitude. BA = Bahamas; CU = Cuba; NI = Nicaragua; FL = Florida; DR = Dominican Republic; MX = Mexico; HO = Honduras; HA = Haiti; BE = Belize; VE = Venezuela; JA = Jamaica; TC = Turks and Caicos; CO = Columbia; PA = Panama; CR = Costa Rica; CA = Cayman Islands; PR = Puerto Rico; LW = Leeward Islands (10 countries); WW = Windward Islands (9 countries); ABC = Aruba, Bonaire, and Curacao. Figure S21 in File S1. A comparison of possible larval sources to the Mexican Quintana Roo coast between two Lagrangian individual based models. The origins of larvae that arrived to habitat on the Quintana Roo coast during April, May, September, and October. Results from Briones-Fourzan are averages between their figures 10 and 11 [29]. Results from our study, using a simulation with passive larvae released equally in magnitude and timing from around the Caribbean (red), and another incorporating vertical migration behavior and larvae released based on reproductive biology (blue). Figure S22 in File S1. The seasonal pattern of observed postlarval arrival compared to modeled predictions, without considering population structure. A comparison of the actual coastal arrival of P. argus postlarvae (red) as compared to modeled predictions (black) over four years at four different locations (Mexico: Bahia de Ascension, Puerto Morales; Florida: Long Key, Big Munson). The Mexican observations are averages from Briones-Fourzan [29]. The Florida FWC observations [35] are of average postlarval arrivals per collector from 2004–2008. The model parameterization ignored seasonal reproductive characteristics and population sizes. There was no significant (p<0.05) correlation between the modele [file pone.0064970.s003.zip › FileS1/S11.tif]

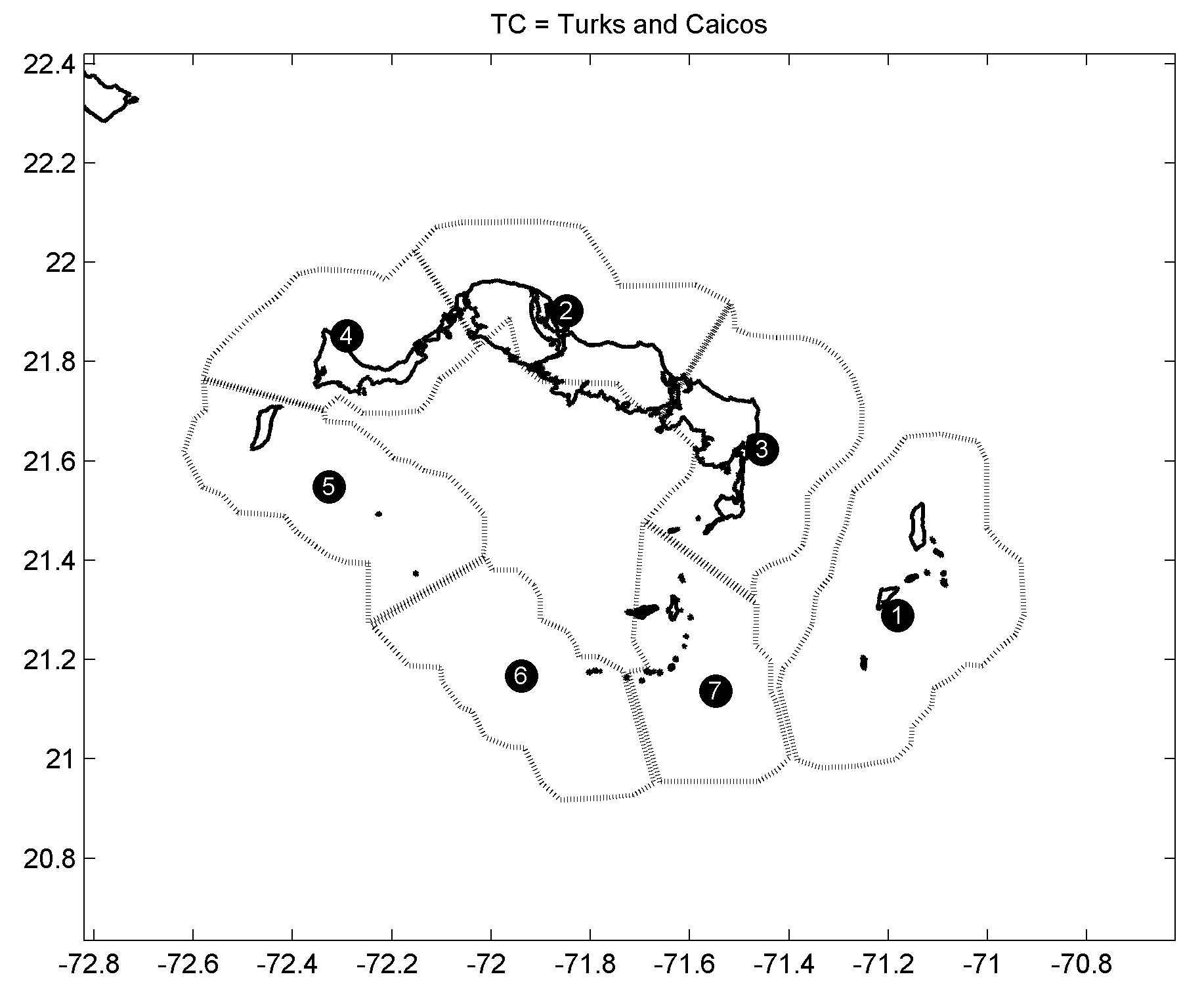

Supplement: File S1 — Contains Figures S1 to S22.: Figure S1 to S20 in File S1. Habitat maps used for the simulation. For each country the habitat sites are shown. Sites within each country are numbered according to the location on the previous figures (2 and 3) reading from left to right along the X axis. All axes are latitude and longitude. BA = Bahamas; CU = Cuba; NI = Nicaragua; FL = Florida; DR = Dominican Republic; MX = Mexico; HO = Honduras; HA = Haiti; BE = Belize; VE = Venezuela; JA = Jamaica; TC = Turks and Caicos; CO = Columbia; PA = Panama; CR = Costa Rica; CA = Cayman Islands; PR = Puerto Rico; LW = Leeward Islands (10 countries); WW = Windward Islands (9 countries); ABC = Aruba, Bonaire, and Curacao. Figure S21 in File S1. A comparison of possible larval sources to the Mexican Quintana Roo coast between two Lagrangian individual based models. The origins of larvae that arrived to habitat on the Quintana Roo coast during April, May, September, and October. Results from Briones-Fourzan are averages between their figures 10 and 11 [29]. Results from our study, using a simulation with passive larvae released equally in magnitude and timing from around the Caribbean (red), and another incorporating vertical migration behavior and larvae released based on reproductive biology (blue). Figure S22 in File S1. The seasonal pattern of observed postlarval arrival compared to modeled predictions, without considering population structure. A comparison of the actual coastal arrival of P. argus postlarvae (red) as compared to modeled predictions (black) over four years at four different locations (Mexico: Bahia de Ascension, Puerto Morales; Florida: Long Key, Big Munson). The Mexican observations are averages from Briones-Fourzan [29]. The Florida FWC observations [35] are of average postlarval arrivals per collector from 2004–2008. The model parameterization ignored seasonal reproductive characteristics and population sizes. There was no significant (p<0.05) correlation between the modele [file pone.0064970.s003.zip › FileS1/S12.tif]

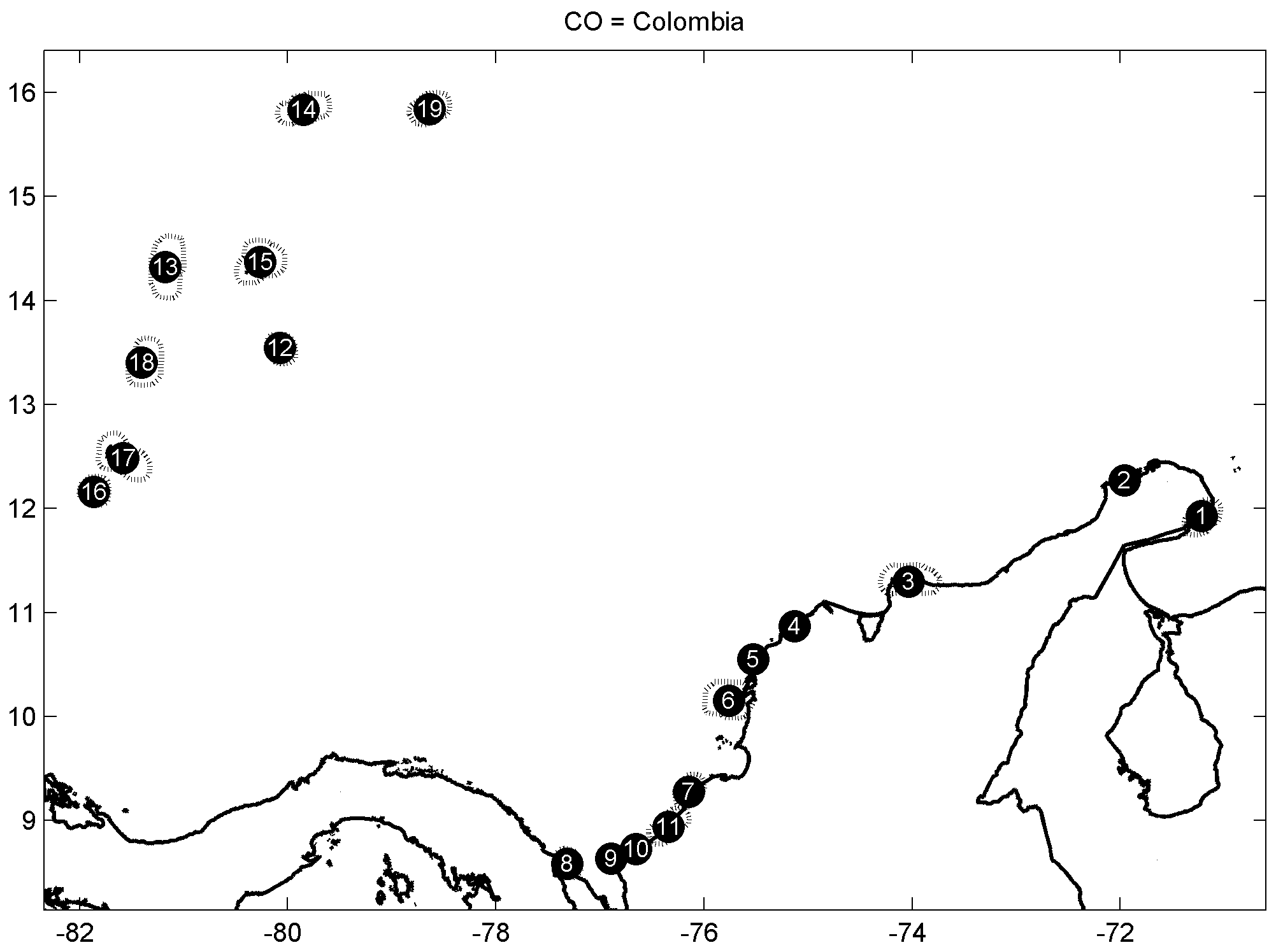

Supplement: File S1 — Contains Figures S1 to S22.: Figure S1 to S20 in File S1. Habitat maps used for the simulation. For each country the habitat sites are shown. Sites within each country are numbered according to the location on the previous figures (2 and 3) reading from left to right along the X axis. All axes are latitude and longitude. BA = Bahamas; CU = Cuba; NI = Nicaragua; FL = Florida; DR = Dominican Republic; MX = Mexico; HO = Honduras; HA = Haiti; BE = Belize; VE = Venezuela; JA = Jamaica; TC = Turks and Caicos; CO = Columbia; PA = Panama; CR = Costa Rica; CA = Cayman Islands; PR = Puerto Rico; LW = Leeward Islands (10 countries); WW = Windward Islands (9 countries); ABC = Aruba, Bonaire, and Curacao. Figure S21 in File S1. A comparison of possible larval sources to the Mexican Quintana Roo coast between two Lagrangian individual based models. The origins of larvae that arrived to habitat on the Quintana Roo coast during April, May, September, and October. Results from Briones-Fourzan are averages between their figures 10 and 11 [29]. Results from our study, using a simulation with passive larvae released equally in magnitude and timing from around the Caribbean (red), and another incorporating vertical migration behavior and larvae released based on reproductive biology (blue). Figure S22 in File S1. The seasonal pattern of observed postlarval arrival compared to modeled predictions, without considering population structure. A comparison of the actual coastal arrival of P. argus postlarvae (red) as compared to modeled predictions (black) over four years at four different locations (Mexico: Bahia de Ascension, Puerto Morales; Florida: Long Key, Big Munson). The Mexican observations are averages from Briones-Fourzan [29]. The Florida FWC observations [35] are of average postlarval arrivals per collector from 2004–2008. The model parameterization ignored seasonal reproductive characteristics and population sizes. There was no significant (p<0.05) correlation between the modele [file pone.0064970.s003.zip › FileS1/S13.tif]

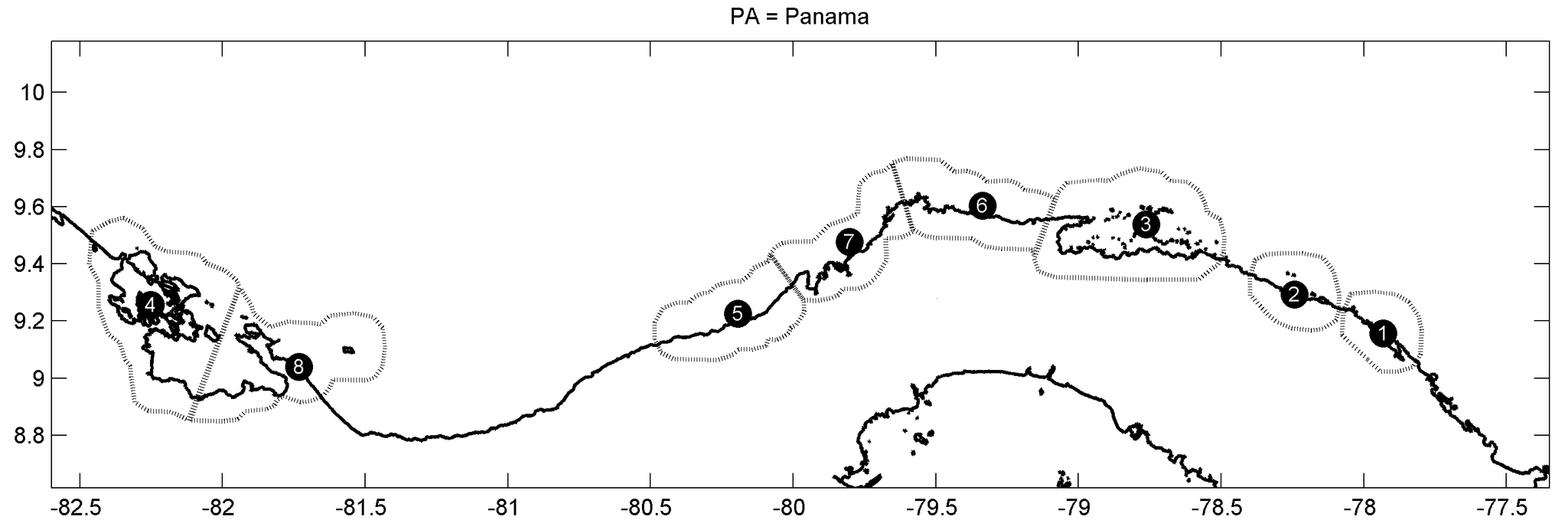

Supplement: File S1 — Contains Figures S1 to S22.: Figure S1 to S20 in File S1. Habitat maps used for the simulation. For each country the habitat sites are shown. Sites within each country are numbered according to the location on the previous figures (2 and 3) reading from left to right along the X axis. All axes are latitude and longitude. BA = Bahamas; CU = Cuba; NI = Nicaragua; FL = Florida; DR = Dominican Republic; MX = Mexico; HO = Honduras; HA = Haiti; BE = Belize; VE = Venezuela; JA = Jamaica; TC = Turks and Caicos; CO = Columbia; PA = Panama; CR = Costa Rica; CA = Cayman Islands; PR = Puerto Rico; LW = Leeward Islands (10 countries); WW = Windward Islands (9 countries); ABC = Aruba, Bonaire, and Curacao. Figure S21 in File S1. A comparison of possible larval sources to the Mexican Quintana Roo coast between two Lagrangian individual based models. The origins of larvae that arrived to habitat on the Quintana Roo coast during April, May, September, and October. Results from Briones-Fourzan are averages between their figures 10 and 11 [29]. Results from our study, using a simulation with passive larvae released equally in magnitude and timing from around the Caribbean (red), and another incorporating vertical migration behavior and larvae released based on reproductive biology (blue). Figure S22 in File S1. The seasonal pattern of observed postlarval arrival compared to modeled predictions, without considering population structure. A comparison of the actual coastal arrival of P. argus postlarvae (red) as compared to modeled predictions (black) over four years at four different locations (Mexico: Bahia de Ascension, Puerto Morales; Florida: Long Key, Big Munson). The Mexican observations are averages from Briones-Fourzan [29]. The Florida FWC observations [35] are of average postlarval arrivals per collector from 2004–2008. The model parameterization ignored seasonal reproductive characteristics and population sizes. There was no significant (p<0.05) correlation between the modele [file pone.0064970.s003.zip › FileS1/S14.tif]

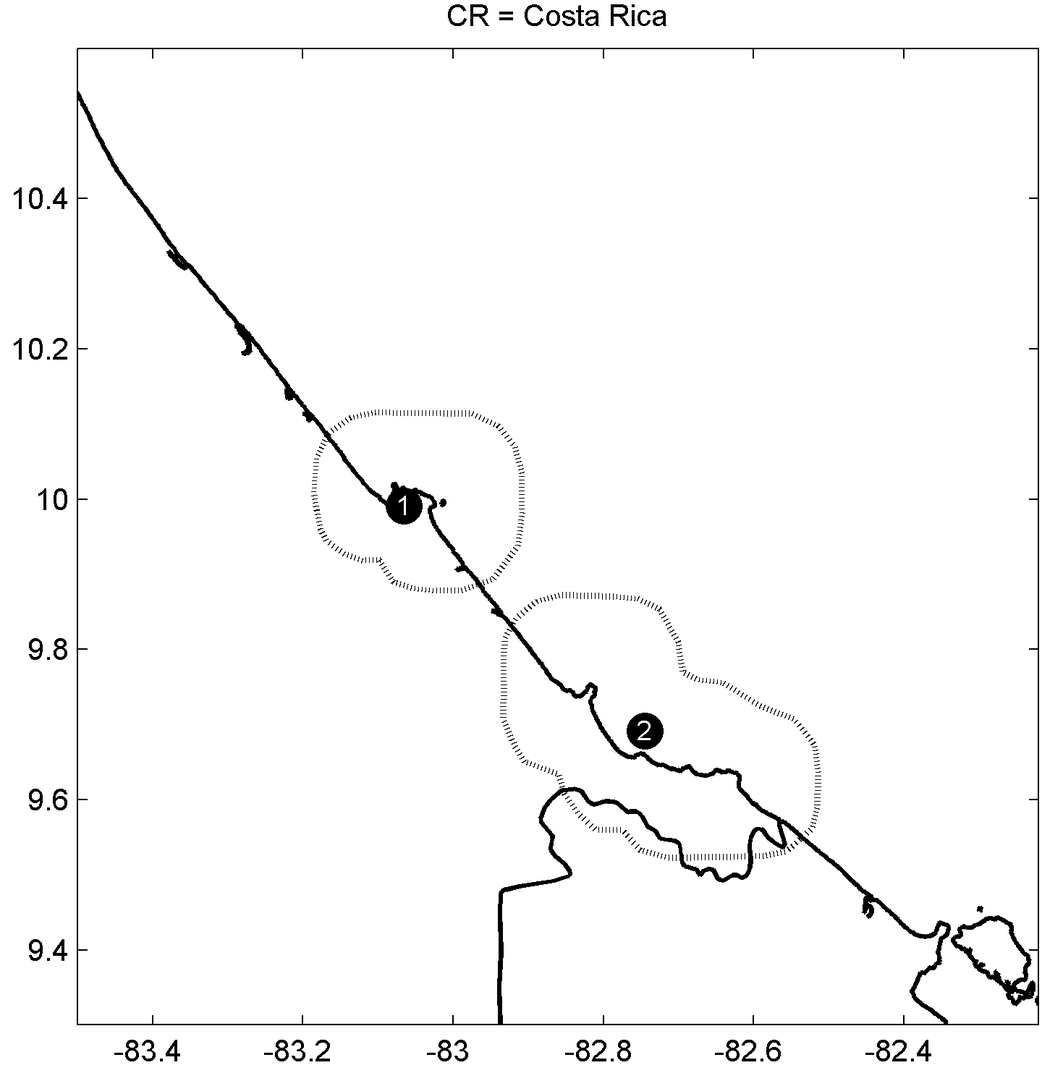

Supplement: File S1 — Contains Figures S1 to S22.: Figure S1 to S20 in File S1. Habitat maps used for the simulation. For each country the habitat sites are shown. Sites within each country are numbered according to the location on the previous figures (2 and 3) reading from left to right along the X axis. All axes are latitude and longitude. BA = Bahamas; CU = Cuba; NI = Nicaragua; FL = Florida; DR = Dominican Republic; MX = Mexico; HO = Honduras; HA = Haiti; BE = Belize; VE = Venezuela; JA = Jamaica; TC = Turks and Caicos; CO = Columbia; PA = Panama; CR = Costa Rica; CA = Cayman Islands; PR = Puerto Rico; LW = Leeward Islands (10 countries); WW = Windward Islands (9 countries); ABC = Aruba, Bonaire, and Curacao. Figure S21 in File S1. A comparison of possible larval sources to the Mexican Quintana Roo coast between two Lagrangian individual based models. The origins of larvae that arrived to habitat on the Quintana Roo coast during April, May, September, and October. Results from Briones-Fourzan are averages between their figures 10 and 11 [29]. Results from our study, using a simulation with passive larvae released equally in magnitude and timing from around the Caribbean (red), and another incorporating vertical migration behavior and larvae released based on reproductive biology (blue). Figure S22 in File S1. The seasonal pattern of observed postlarval arrival compared to modeled predictions, without considering population structure. A comparison of the actual coastal arrival of P. argus postlarvae (red) as compared to modeled predictions (black) over four years at four different locations (Mexico: Bahia de Ascension, Puerto Morales; Florida: Long Key, Big Munson). The Mexican observations are averages from Briones-Fourzan [29]. The Florida FWC observations [35] are of average postlarval arrivals per collector from 2004–2008. The model parameterization ignored seasonal reproductive characteristics and population sizes. There was no significant (p<0.05) correlation between the modele [file pone.0064970.s003.zip › FileS1/S15.tif]

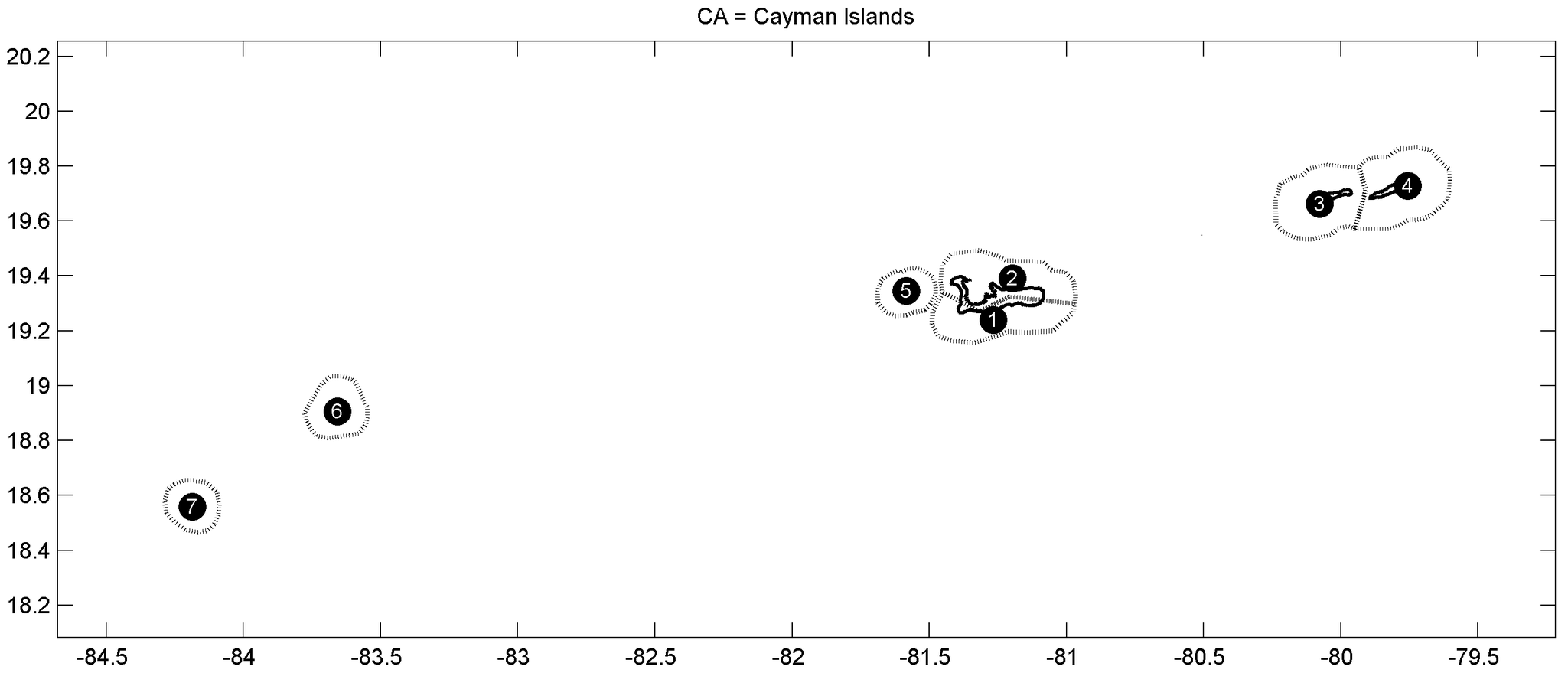

Supplement: File S1 — Contains Figures S1 to S22.: Figure S1 to S20 in File S1. Habitat maps used for the simulation. For each country the habitat sites are shown. Sites within each country are numbered according to the location on the previous figures (2 and 3) reading from left to right along the X axis. All axes are latitude and longitude. BA = Bahamas; CU = Cuba; NI = Nicaragua; FL = Florida; DR = Dominican Republic; MX = Mexico; HO = Honduras; HA = Haiti; BE = Belize; VE = Venezuela; JA = Jamaica; TC = Turks and Caicos; CO = Columbia; PA = Panama; CR = Costa Rica; CA = Cayman Islands; PR = Puerto Rico; LW = Leeward Islands (10 countries); WW = Windward Islands (9 countries); ABC = Aruba, Bonaire, and Curacao. Figure S21 in File S1. A comparison of possible larval sources to the Mexican Quintana Roo coast between two Lagrangian individual based models. The origins of larvae that arrived to habitat on the Quintana Roo coast during April, May, September, and October. Results from Briones-Fourzan are averages between their figures 10 and 11 [29]. Results from our study, using a simulation with passive larvae released equally in magnitude and timing from around the Caribbean (red), and another incorporating vertical migration behavior and larvae released based on reproductive biology (blue). Figure S22 in File S1. The seasonal pattern of observed postlarval arrival compared to modeled predictions, without considering population structure. A comparison of the actual coastal arrival of P. argus postlarvae (red) as compared to modeled predictions (black) over four years at four different locations (Mexico: Bahia de Ascension, Puerto Morales; Florida: Long Key, Big Munson). The Mexican observations are averages from Briones-Fourzan [29]. The Florida FWC observations [35] are of average postlarval arrivals per collector from 2004–2008. The model parameterization ignored seasonal reproductive characteristics and population sizes. There was no significant (p<0.05) correlation between the modele [file pone.0064970.s003.zip › FileS1/S16.tif]

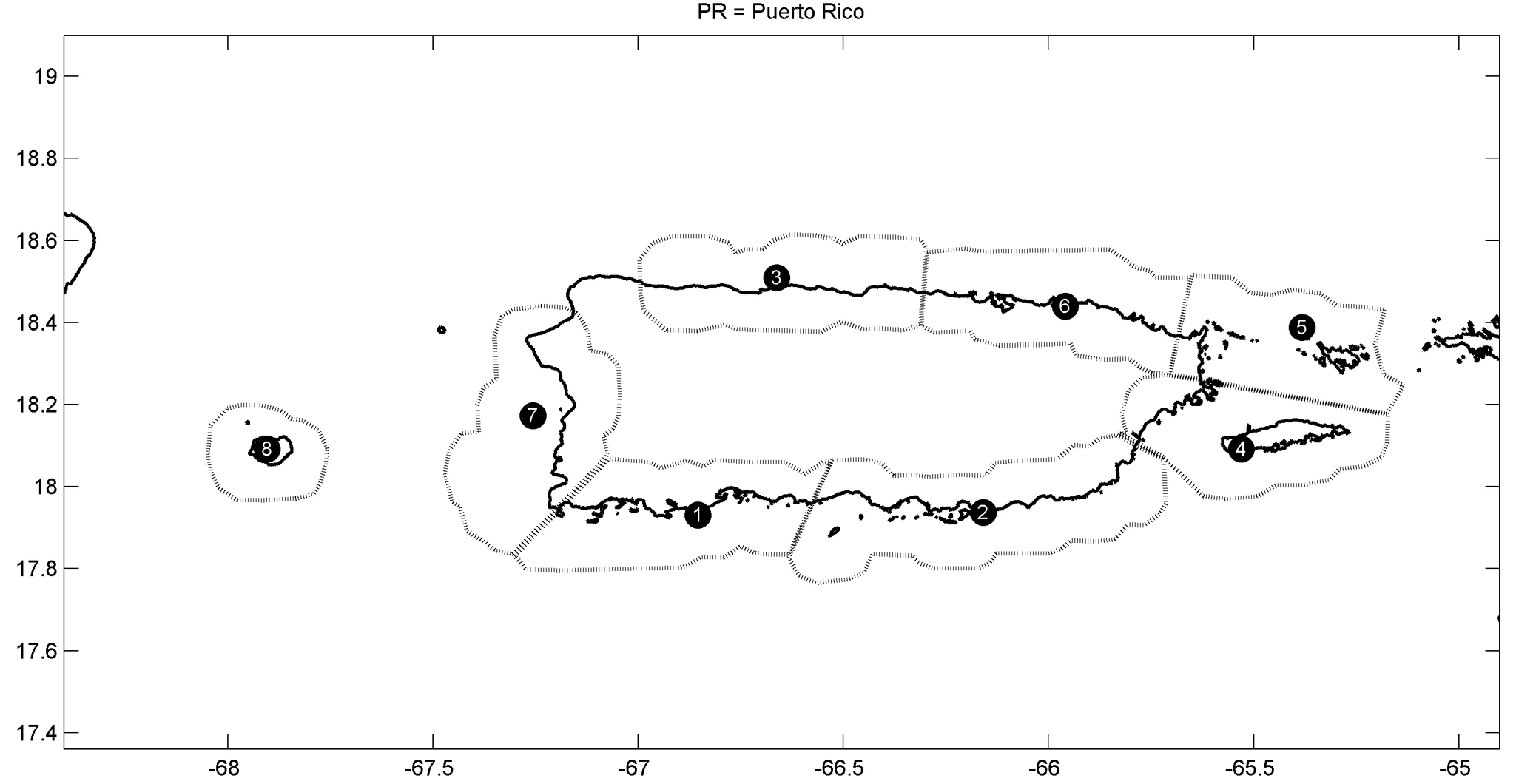

Supplement: File S1 — Contains Figures S1 to S22.: Figure S1 to S20 in File S1. Habitat maps used for the simulation. For each country the habitat sites are shown. Sites within each country are numbered according to the location on the previous figures (2 and 3) reading from left to right along the X axis. All axes are latitude and longitude. BA = Bahamas; CU = Cuba; NI = Nicaragua; FL = Florida; DR = Dominican Republic; MX = Mexico; HO = Honduras; HA = Haiti; BE = Belize; VE = Venezuela; JA = Jamaica; TC = Turks and Caicos; CO = Columbia; PA = Panama; CR = Costa Rica; CA = Cayman Islands; PR = Puerto Rico; LW = Leeward Islands (10 countries); WW = Windward Islands (9 countries); ABC = Aruba, Bonaire, and Curacao. Figure S21 in File S1. A comparison of possible larval sources to the Mexican Quintana Roo coast between two Lagrangian individual based models. The origins of larvae that arrived to habitat on the Quintana Roo coast during April, May, September, and October. Results from Briones-Fourzan are averages between their figures 10 and 11 [29]. Results from our study, using a simulation with passive larvae released equally in magnitude and timing from around the Caribbean (red), and another incorporating vertical migration behavior and larvae released based on reproductive biology (blue). Figure S22 in File S1. The seasonal pattern of observed postlarval arrival compared to modeled predictions, without considering population structure. A comparison of the actual coastal arrival of P. argus postlarvae (red) as compared to modeled predictions (black) over four years at four different locations (Mexico: Bahia de Ascension, Puerto Morales; Florida: Long Key, Big Munson). The Mexican observations are averages from Briones-Fourzan [29]. The Florida FWC observations [35] are of average postlarval arrivals per collector from 2004–2008. The model parameterization ignored seasonal reproductive characteristics and population sizes. There was no significant (p<0.05) correlation between the modele [file pone.0064970.s003.zip › FileS1/S17.tif]

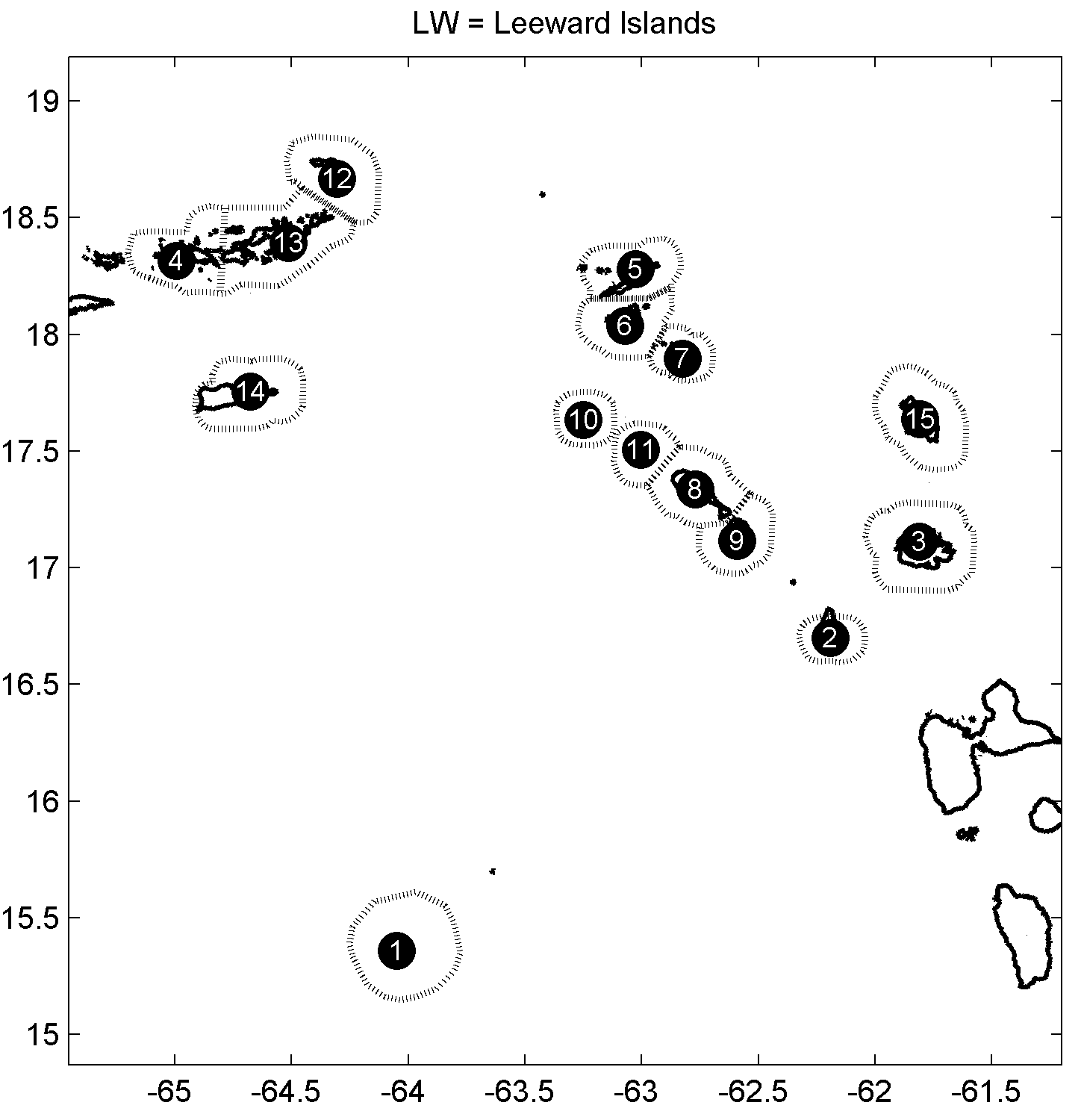

Supplement: File S1 — Contains Figures S1 to S22.: Figure S1 to S20 in File S1. Habitat maps used for the simulation. For each country the habitat sites are shown. Sites within each country are numbered according to the location on the previous figures (2 and 3) reading from left to right along the X axis. All axes are latitude and longitude. BA = Bahamas; CU = Cuba; NI = Nicaragua; FL = Florida; DR = Dominican Republic; MX = Mexico; HO = Honduras; HA = Haiti; BE = Belize; VE = Venezuela; JA = Jamaica; TC = Turks and Caicos; CO = Columbia; PA = Panama; CR = Costa Rica; CA = Cayman Islands; PR = Puerto Rico; LW = Leeward Islands (10 countries); WW = Windward Islands (9 countries); ABC = Aruba, Bonaire, and Curacao. Figure S21 in File S1. A comparison of possible larval sources to the Mexican Quintana Roo coast between two Lagrangian individual based models. The origins of larvae that arrived to habitat on the Quintana Roo coast during April, May, September, and October. Results from Briones-Fourzan are averages between their figures 10 and 11 [29]. Results from our study, using a simulation with passive larvae released equally in magnitude and timing from around the Caribbean (red), and another incorporating vertical migration behavior and larvae released based on reproductive biology (blue). Figure S22 in File S1. The seasonal pattern of observed postlarval arrival compared to modeled predictions, without considering population structure. A comparison of the actual coastal arrival of P. argus postlarvae (red) as compared to modeled predictions (black) over four years at four different locations (Mexico: Bahia de Ascension, Puerto Morales; Florida: Long Key, Big Munson). The Mexican observations are averages from Briones-Fourzan [29]. The Florida FWC observations [35] are of average postlarval arrivals per collector from 2004–2008. The model parameterization ignored seasonal reproductive characteristics and population sizes. There was no significant (p<0.05) correlation between the modele [file pone.0064970.s003.zip › FileS1/S18.tif]

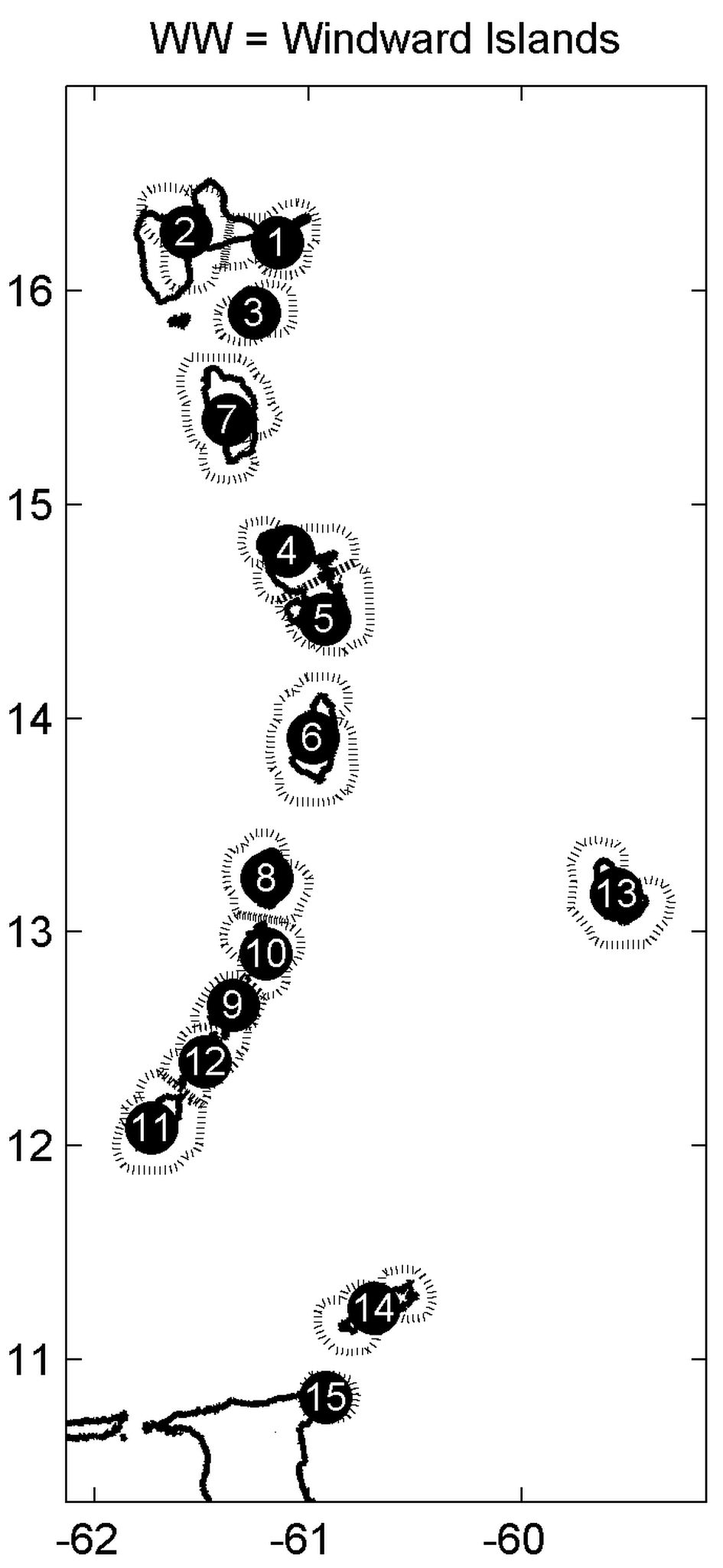

Supplement: File S1 — Contains Figures S1 to S22.: Figure S1 to S20 in File S1. Habitat maps used for the simulation. For each country the habitat sites are shown. Sites within each country are numbered according to the location on the previous figures (2 and 3) reading from left to right along the X axis. All axes are latitude and longitude. BA = Bahamas; CU = Cuba; NI = Nicaragua; FL = Florida; DR = Dominican Republic; MX = Mexico; HO = Honduras; HA = Haiti; BE = Belize; VE = Venezuela; JA = Jamaica; TC = Turks and Caicos; CO = Columbia; PA = Panama; CR = Costa Rica; CA = Cayman Islands; PR = Puerto Rico; LW = Leeward Islands (10 countries); WW = Windward Islands (9 countries); ABC = Aruba, Bonaire, and Curacao. Figure S21 in File S1. A comparison of possible larval sources to the Mexican Quintana Roo coast between two Lagrangian individual based models. The origins of larvae that arrived to habitat on the Quintana Roo coast during April, May, September, and October. Results from Briones-Fourzan are averages between their figures 10 and 11 [29]. Results from our study, using a simulation with passive larvae released equally in magnitude and timing from around the Caribbean (red), and another incorporating vertical migration behavior and larvae released based on reproductive biology (blue). Figure S22 in File S1. The seasonal pattern of observed postlarval arrival compared to modeled predictions, without considering population structure. A comparison of the actual coastal arrival of P. argus postlarvae (red) as compared to modeled predictions (black) over four years at four different locations (Mexico: Bahia de Ascension, Puerto Morales; Florida: Long Key, Big Munson). The Mexican observations are averages from Briones-Fourzan [29]. The Florida FWC observations [35] are of average postlarval arrivals per collector from 2004–2008. The model parameterization ignored seasonal reproductive characteristics and population sizes. There was no significant (p<0.05) correlation between the modele [file pone.0064970.s003.zip › FileS1/S19.tif]

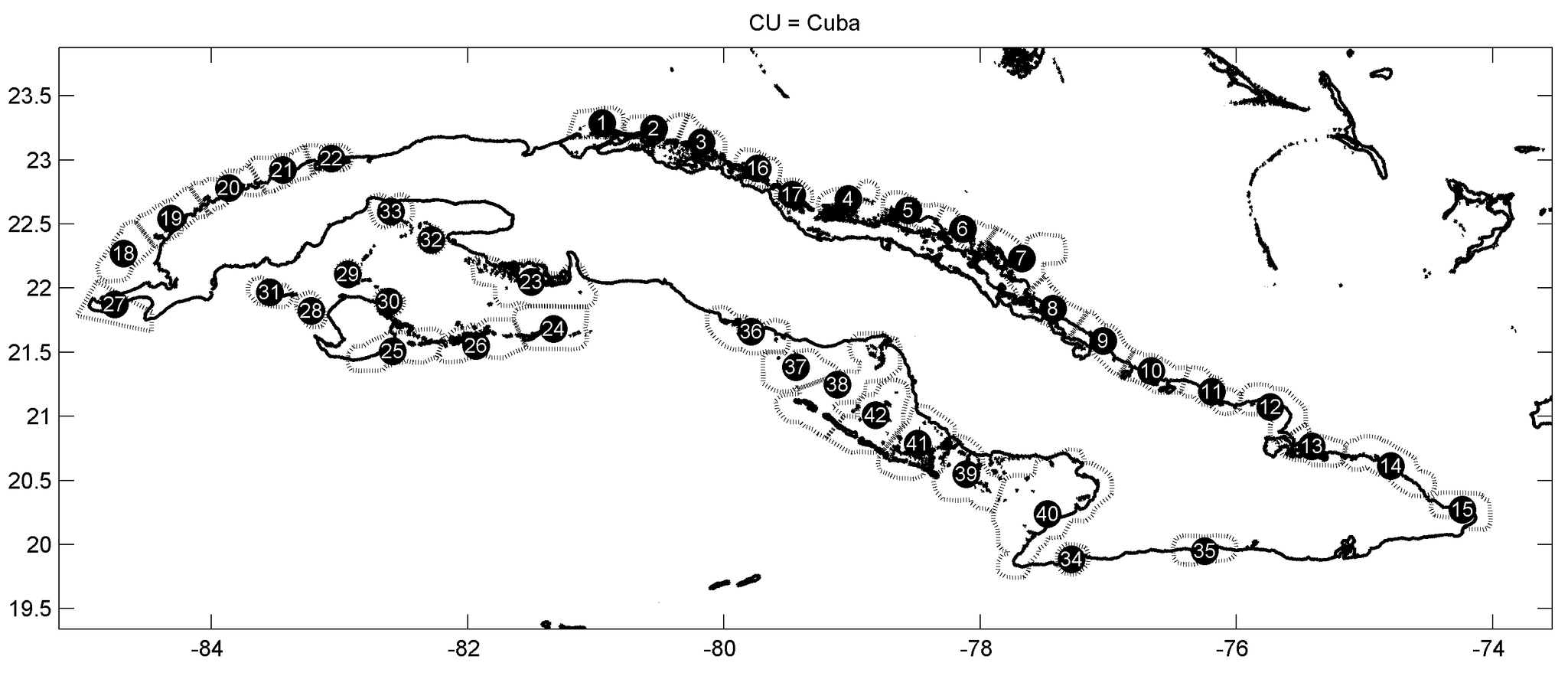

Supplement: File S1 — Contains Figures S1 to S22.: Figure S1 to S20 in File S1. Habitat maps used for the simulation. For each country the habitat sites are shown. Sites within each country are numbered according to the location on the previous figures (2 and 3) reading from left to right along the X axis. All axes are latitude and longitude. BA = Bahamas; CU = Cuba; NI = Nicaragua; FL = Florida; DR = Dominican Republic; MX = Mexico; HO = Honduras; HA = Haiti; BE = Belize; VE = Venezuela; JA = Jamaica; TC = Turks and Caicos; CO = Columbia; PA = Panama; CR = Costa Rica; CA = Cayman Islands; PR = Puerto Rico; LW = Leeward Islands (10 countries); WW = Windward Islands (9 countries); ABC = Aruba, Bonaire, and Curacao. Figure S21 in File S1. A comparison of possible larval sources to the Mexican Quintana Roo coast between two Lagrangian individual based models. The origins of larvae that arrived to habitat on the Quintana Roo coast during April, May, September, and October. Results from Briones-Fourzan are averages between their figures 10 and 11 [29]. Results from our study, using a simulation with passive larvae released equally in magnitude and timing from around the Caribbean (red), and another incorporating vertical migration behavior and larvae released based on reproductive biology (blue). Figure S22 in File S1. The seasonal pattern of observed postlarval arrival compared to modeled predictions, without considering population structure. A comparison of the actual coastal arrival of P. argus postlarvae (red) as compared to modeled predictions (black) over four years at four different locations (Mexico: Bahia de Ascension, Puerto Morales; Florida: Long Key, Big Munson). The Mexican observations are averages from Briones-Fourzan [29]. The Florida FWC observations [35] are of average postlarval arrivals per collector from 2004–2008. The model parameterization ignored seasonal reproductive characteristics and population sizes. There was no significant (p<0.05) correlation between the modele [file pone.0064970.s003.zip › FileS1/S2.tif]

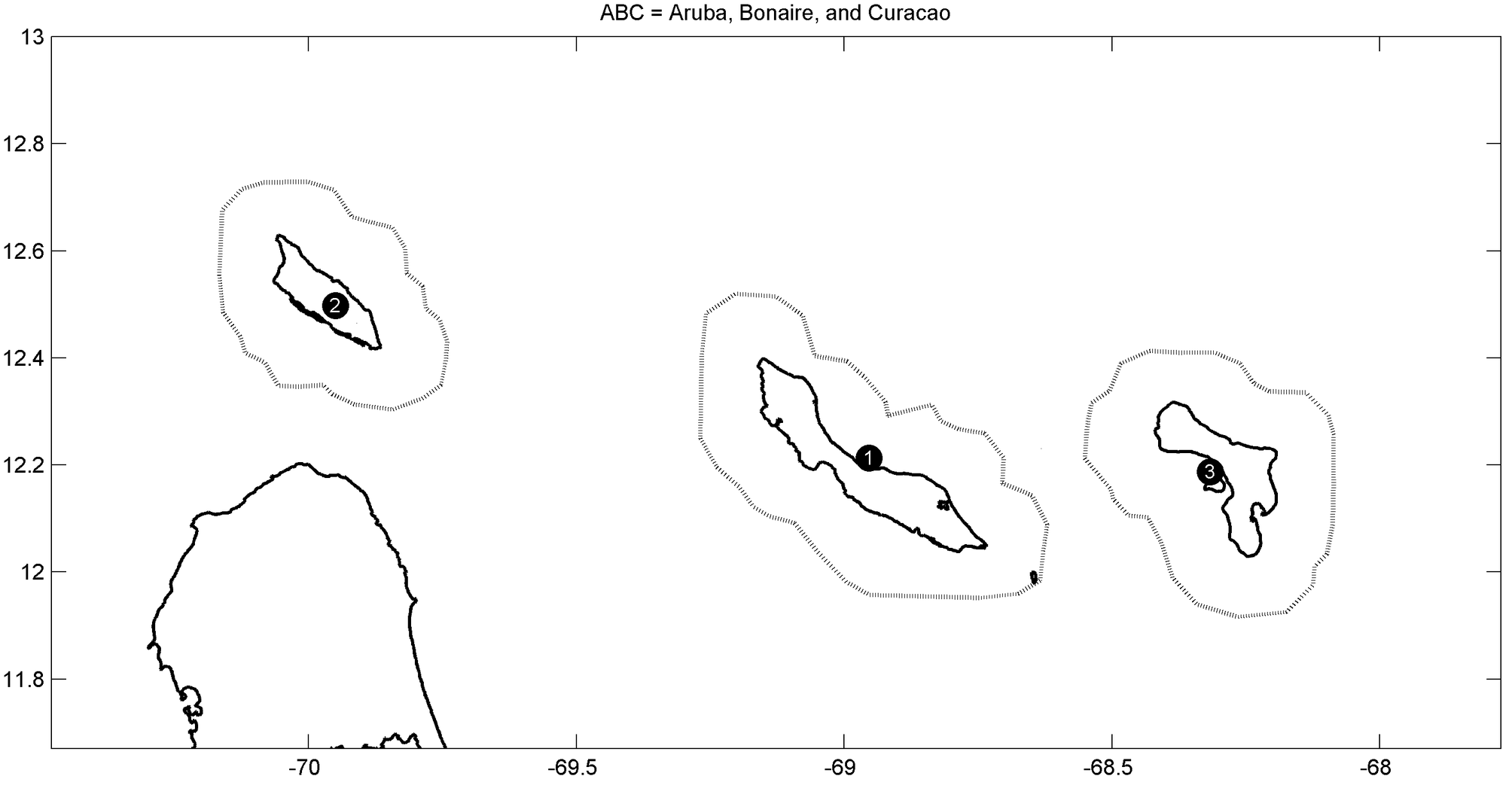

Supplement: File S1 — Contains Figures S1 to S22.: Figure S1 to S20 in File S1. Habitat maps used for the simulation. For each country the habitat sites are shown. Sites within each country are numbered according to the location on the previous figures (2 and 3) reading from left to right along the X axis. All axes are latitude and longitude. BA = Bahamas; CU = Cuba; NI = Nicaragua; FL = Florida; DR = Dominican Republic; MX = Mexico; HO = Honduras; HA = Haiti; BE = Belize; VE = Venezuela; JA = Jamaica; TC = Turks and Caicos; CO = Columbia; PA = Panama; CR = Costa Rica; CA = Cayman Islands; PR = Puerto Rico; LW = Leeward Islands (10 countries); WW = Windward Islands (9 countries); ABC = Aruba, Bonaire, and Curacao. Figure S21 in File S1. A comparison of possible larval sources to the Mexican Quintana Roo coast between two Lagrangian individual based models. The origins of larvae that arrived to habitat on the Quintana Roo coast during April, May, September, and October. Results from Briones-Fourzan are averages between their figures 10 and 11 [29]. Results from our study, using a simulation with passive larvae released equally in magnitude and timing from around the Caribbean (red), and another incorporating vertical migration behavior and larvae released based on reproductive biology (blue). Figure S22 in File S1. The seasonal pattern of observed postlarval arrival compared to modeled predictions, without considering population structure. A comparison of the actual coastal arrival of P. argus postlarvae (red) as compared to modeled predictions (black) over four years at four different locations (Mexico: Bahia de Ascension, Puerto Morales; Florida: Long Key, Big Munson). The Mexican observations are averages from Briones-Fourzan [29]. The Florida FWC observations [35] are of average postlarval arrivals per collector from 2004–2008. The model parameterization ignored seasonal reproductive characteristics and population sizes. There was no significant (p<0.05) correlation between the modele [file pone.0064970.s003.zip › FileS1/S20.tif]

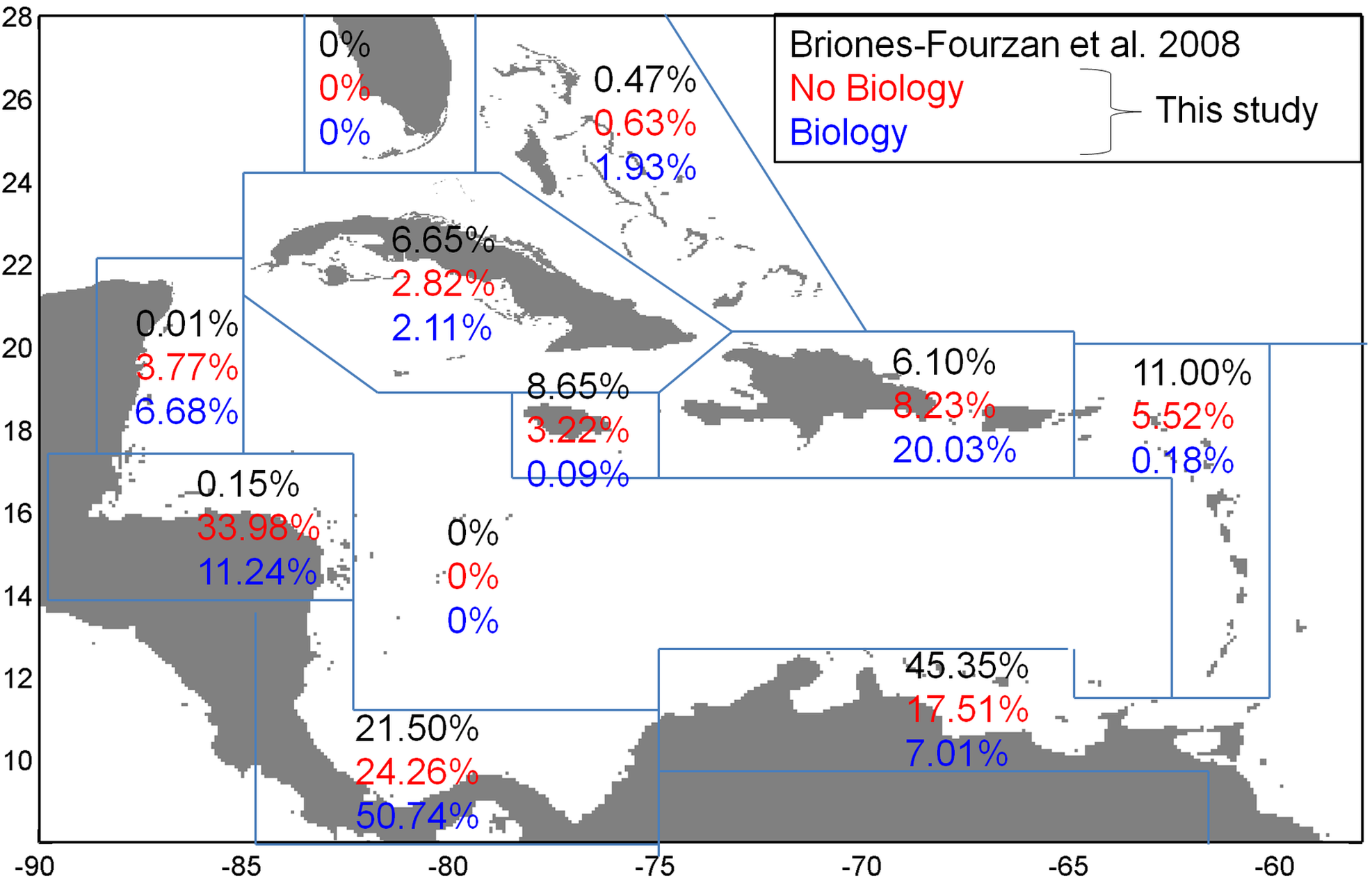

Supplement: File S1 — Contains Figures S1 to S22.: Figure S1 to S20 in File S1. Habitat maps used for the simulation. For each country the habitat sites are shown. Sites within each country are numbered according to the location on the previous figures (2 and 3) reading from left to right along the X axis. All axes are latitude and longitude. BA = Bahamas; CU = Cuba; NI = Nicaragua; FL = Florida; DR = Dominican Republic; MX = Mexico; HO = Honduras; HA = Haiti; BE = Belize; VE = Venezuela; JA = Jamaica; TC = Turks and Caicos; CO = Columbia; PA = Panama; CR = Costa Rica; CA = Cayman Islands; PR = Puerto Rico; LW = Leeward Islands (10 countries); WW = Windward Islands (9 countries); ABC = Aruba, Bonaire, and Curacao. Figure S21 in File S1. A comparison of possible larval sources to the Mexican Quintana Roo coast between two Lagrangian individual based models. The origins of larvae that arrived to habitat on the Quintana Roo coast during April, May, September, and October. Results from Briones-Fourzan are averages between their figures 10 and 11 [29]. Results from our study, using a simulation with passive larvae released equally in magnitude and timing from around the Caribbean (red), and another incorporating vertical migration behavior and larvae released based on reproductive biology (blue). Figure S22 in File S1. The seasonal pattern of observed postlarval arrival compared to modeled predictions, without considering population structure. A comparison of the actual coastal arrival of P. argus postlarvae (red) as compared to modeled predictions (black) over four years at four different locations (Mexico: Bahia de Ascension, Puerto Morales; Florida: Long Key, Big Munson). The Mexican observations are averages from Briones-Fourzan [29]. The Florida FWC observations [35] are of average postlarval arrivals per collector from 2004–2008. The model parameterization ignored seasonal reproductive characteristics and population sizes. There was no significant (p<0.05) correlation between the modele [file pone.0064970.s003.zip › FileS1/S21.tif]

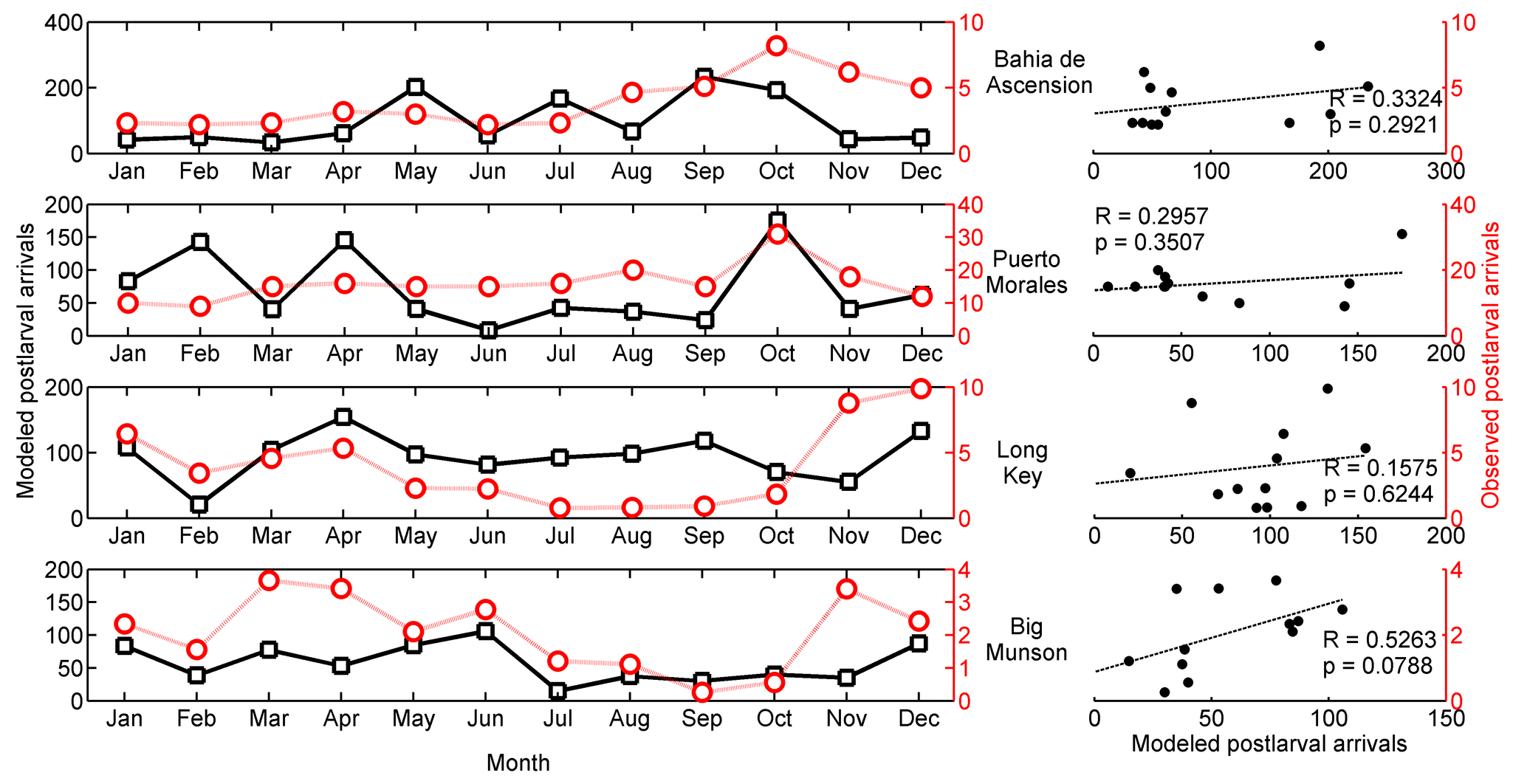

Supplement: File S1 — Contains Figures S1 to S22.: Figure S1 to S20 in File S1. Habitat maps used for the simulation. For each country the habitat sites are shown. Sites within each country are numbered according to the location on the previous figures (2 and 3) reading from left to right along the X axis. All axes are latitude and longitude. BA = Bahamas; CU = Cuba; NI = Nicaragua; FL = Florida; DR = Dominican Republic; MX = Mexico; HO = Honduras; HA = Haiti; BE = Belize; VE = Venezuela; JA = Jamaica; TC = Turks and Caicos; CO = Columbia; PA = Panama; CR = Costa Rica; CA = Cayman Islands; PR = Puerto Rico; LW = Leeward Islands (10 countries); WW = Windward Islands (9 countries); ABC = Aruba, Bonaire, and Curacao. Figure S21 in File S1. A comparison of possible larval sources to the Mexican Quintana Roo coast between two Lagrangian individual based models. The origins of larvae that arrived to habitat on the Quintana Roo coast during April, May, September, and October. Results from Briones-Fourzan are averages between their figures 10 and 11 [29]. Results from our study, using a simulation with passive larvae released equally in magnitude and timing from around the Caribbean (red), and another incorporating vertical migration behavior and larvae released based on reproductive biology (blue). Figure S22 in File S1. The seasonal pattern of observed postlarval arrival compared to modeled predictions, without considering population structure. A comparison of the actual coastal arrival of P. argus postlarvae (red) as compared to modeled predictions (black) over four years at four different locations (Mexico: Bahia de Ascension, Puerto Morales; Florida: Long Key, Big Munson). The Mexican observations are averages from Briones-Fourzan [29]. The Florida FWC observations [35] are of average postlarval arrivals per collector from 2004–2008. The model parameterization ignored seasonal reproductive characteristics and population sizes. There was no significant (p<0.05) correlation between the modele [file pone.0064970.s003.zip › FileS1/S22.tif]

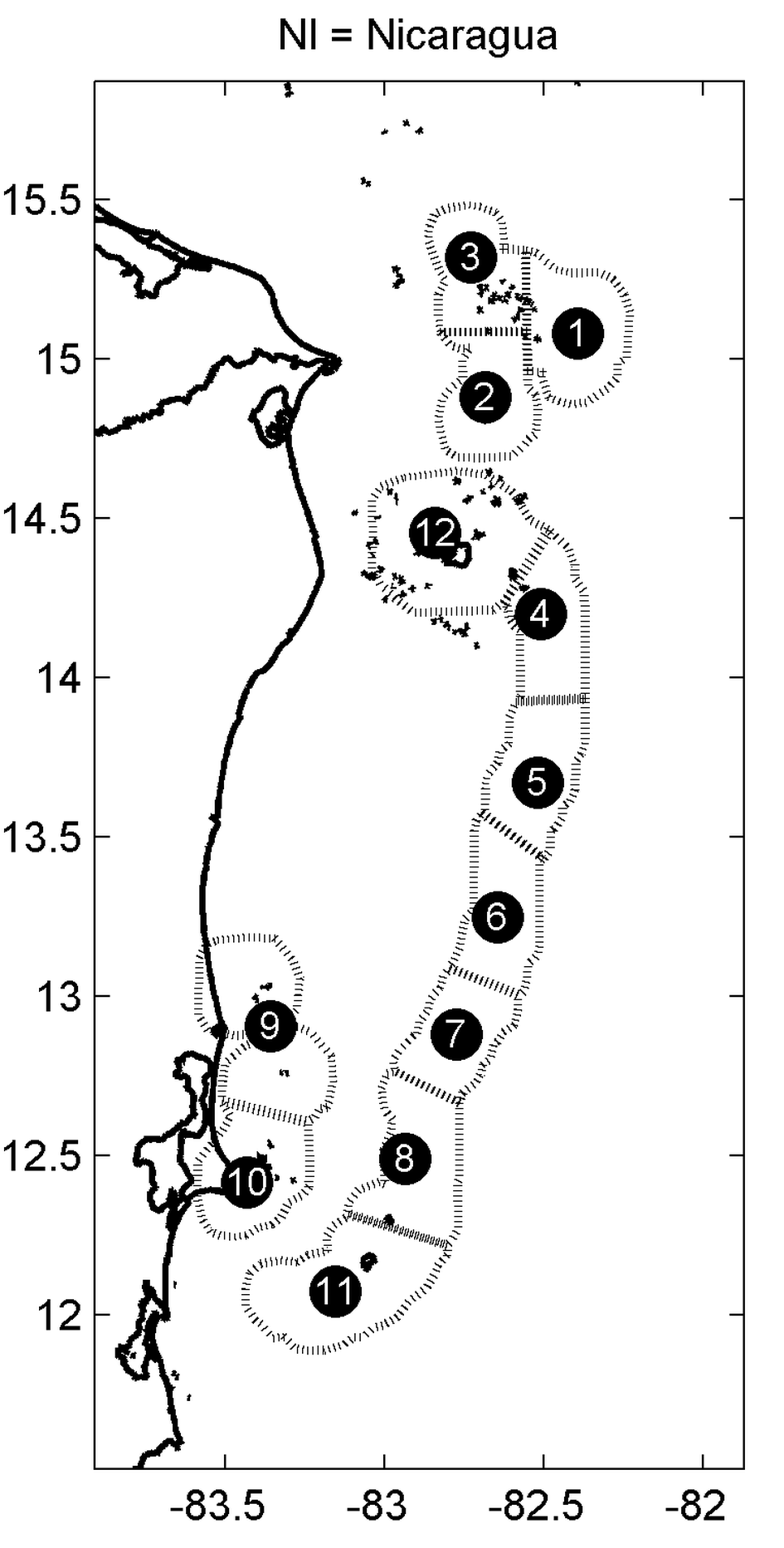

Supplement: File S1 — Contains Figures S1 to S22.: Figure S1 to S20 in File S1. Habitat maps used for the simulation. For each country the habitat sites are shown. Sites within each country are numbered according to the location on the previous figures (2 and 3) reading from left to right along the X axis. All axes are latitude and longitude. BA = Bahamas; CU = Cuba; NI = Nicaragua; FL = Florida; DR = Dominican Republic; MX = Mexico; HO = Honduras; HA = Haiti; BE = Belize; VE = Venezuela; JA = Jamaica; TC = Turks and Caicos; CO = Columbia; PA = Panama; CR = Costa Rica; CA = Cayman Islands; PR = Puerto Rico; LW = Leeward Islands (10 countries); WW = Windward Islands (9 countries); ABC = Aruba, Bonaire, and Curacao. Figure S21 in File S1. A comparison of possible larval sources to the Mexican Quintana Roo coast between two Lagrangian individual based models. The origins of larvae that arrived to habitat on the Quintana Roo coast during April, May, September, and October. Results from Briones-Fourzan are averages between their figures 10 and 11 [29]. Results from our study, using a simulation with passive larvae released equally in magnitude and timing from around the Caribbean (red), and another incorporating vertical migration behavior and larvae released based on reproductive biology (blue). Figure S22 in File S1. The seasonal pattern of observed postlarval arrival compared to modeled predictions, without considering population structure. A comparison of the actual coastal arrival of P. argus postlarvae (red) as compared to modeled predictions (black) over four years at four different locations (Mexico: Bahia de Ascension, Puerto Morales; Florida: Long Key, Big Munson). The Mexican observations are averages from Briones-Fourzan [29]. The Florida FWC observations [35] are of average postlarval arrivals per collector from 2004–2008. The model parameterization ignored seasonal reproductive characteristics and population sizes. There was no significant (p<0.05) correlation between the modele [file pone.0064970.s003.zip › FileS1/S3.tif]

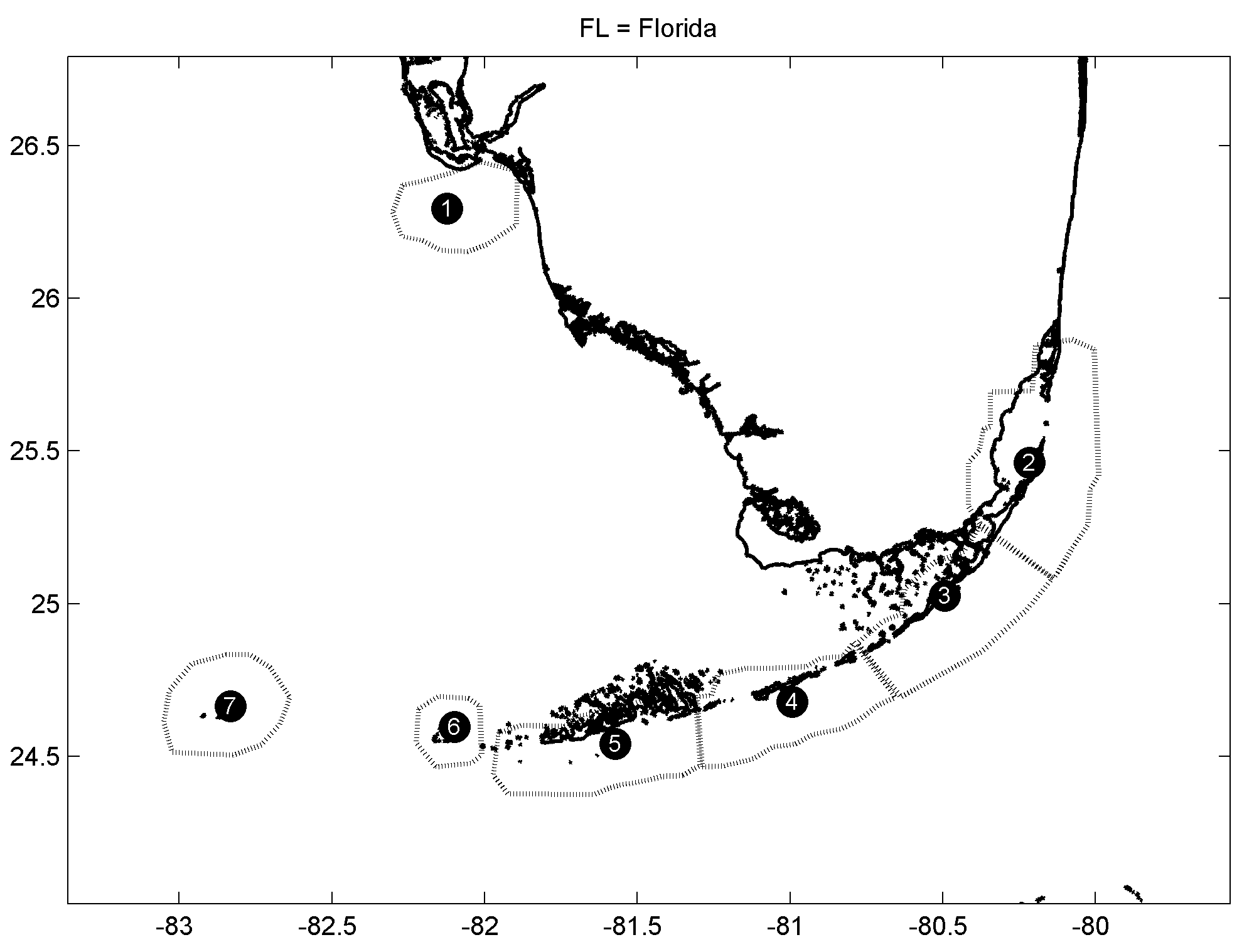

Supplement: File S1 — Contains Figures S1 to S22.: Figure S1 to S20 in File S1. Habitat maps used for the simulation. For each country the habitat sites are shown. Sites within each country are numbered according to the location on the previous figures (2 and 3) reading from left to right along the X axis. All axes are latitude and longitude. BA = Bahamas; CU = Cuba; NI = Nicaragua; FL = Florida; DR = Dominican Republic; MX = Mexico; HO = Honduras; HA = Haiti; BE = Belize; VE = Venezuela; JA = Jamaica; TC = Turks and Caicos; CO = Columbia; PA = Panama; CR = Costa Rica; CA = Cayman Islands; PR = Puerto Rico; LW = Leeward Islands (10 countries); WW = Windward Islands (9 countries); ABC = Aruba, Bonaire, and Curacao. Figure S21 in File S1. A comparison of possible larval sources to the Mexican Quintana Roo coast between two Lagrangian individual based models. The origins of larvae that arrived to habitat on the Quintana Roo coast during April, May, September, and October. Results from Briones-Fourzan are averages between their figures 10 and 11 [29]. Results from our study, using a simulation with passive larvae released equally in magnitude and timing from around the Caribbean (red), and another incorporating vertical migration behavior and larvae released based on reproductive biology (blue). Figure S22 in File S1. The seasonal pattern of observed postlarval arrival compared to modeled predictions, without considering population structure. A comparison of the actual coastal arrival of P. argus postlarvae (red) as compared to modeled predictions (black) over four years at four different locations (Mexico: Bahia de Ascension, Puerto Morales; Florida: Long Key, Big Munson). The Mexican observations are averages from Briones-Fourzan [29]. The Florida FWC observations [35] are of average postlarval arrivals per collector from 2004–2008. The model parameterization ignored seasonal reproductive characteristics and population sizes. There was no significant (p<0.05) correlation between the modele [file pone.0064970.s003.zip › FileS1/S4.tif]

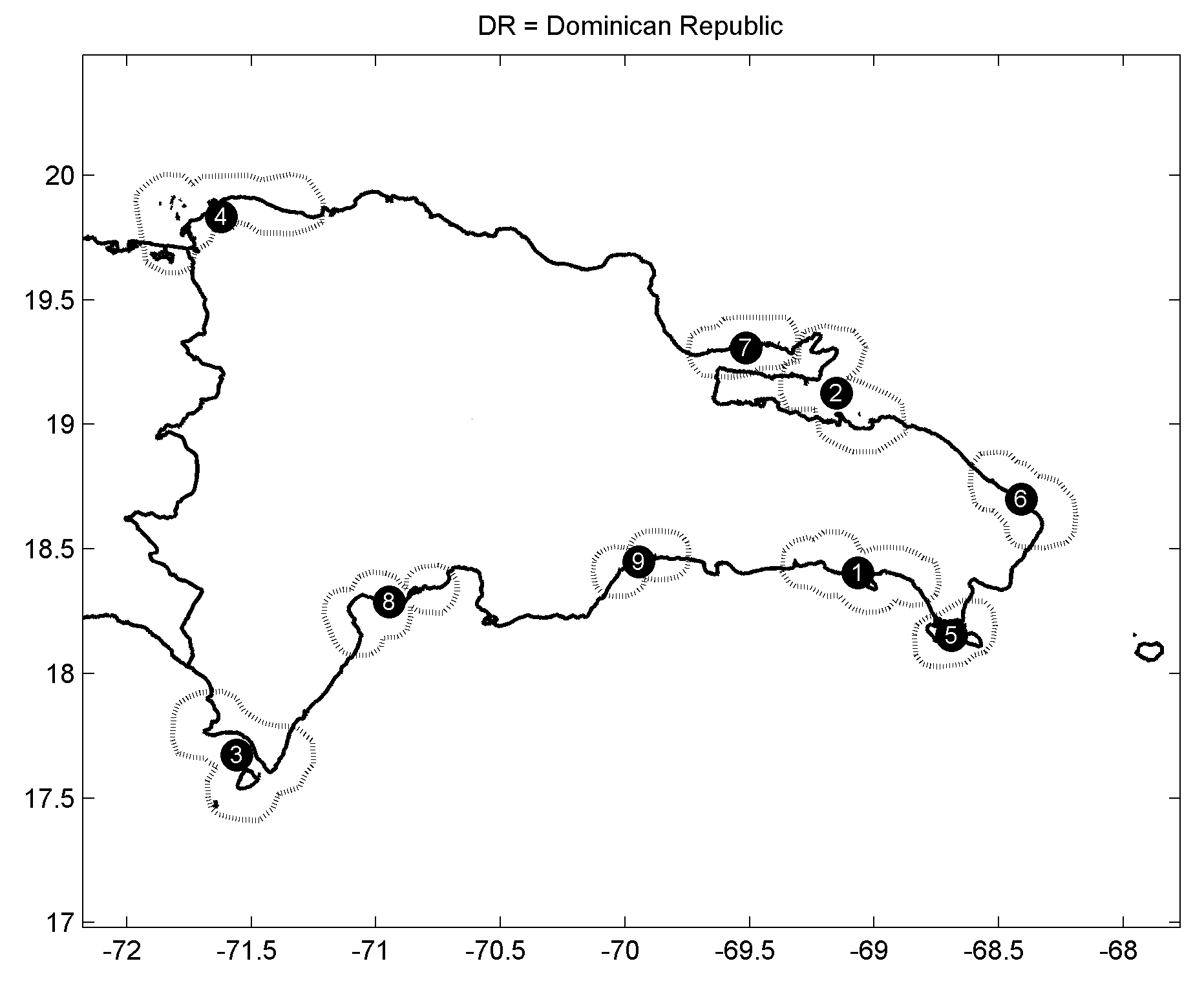

Supplement: File S1 — Contains Figures S1 to S22.: Figure S1 to S20 in File S1. Habitat maps used for the simulation. For each country the habitat sites are shown. Sites within each country are numbered according to the location on the previous figures (2 and 3) reading from left to right along the X axis. All axes are latitude and longitude. BA = Bahamas; CU = Cuba; NI = Nicaragua; FL = Florida; DR = Dominican Republic; MX = Mexico; HO = Honduras; HA = Haiti; BE = Belize; VE = Venezuela; JA = Jamaica; TC = Turks and Caicos; CO = Columbia; PA = Panama; CR = Costa Rica; CA = Cayman Islands; PR = Puerto Rico; LW = Leeward Islands (10 countries); WW = Windward Islands (9 countries); ABC = Aruba, Bonaire, and Curacao. Figure S21 in File S1. A comparison of possible larval sources to the Mexican Quintana Roo coast between two Lagrangian individual based models. The origins of larvae that arrived to habitat on the Quintana Roo coast during April, May, September, and October. Results from Briones-Fourzan are averages between their figures 10 and 11 [29]. Results from our study, using a simulation with passive larvae released equally in magnitude and timing from around the Caribbean (red), and another incorporating vertical migration behavior and larvae released based on reproductive biology (blue). Figure S22 in File S1. The seasonal pattern of observed postlarval arrival compared to modeled predictions, without considering population structure. A comparison of the actual coastal arrival of P. argus postlarvae (red) as compared to modeled predictions (black) over four years at four different locations (Mexico: Bahia de Ascension, Puerto Morales; Florida: Long Key, Big Munson). The Mexican observations are averages from Briones-Fourzan [29]. The Florida FWC observations [35] are of average postlarval arrivals per collector from 2004–2008. The model parameterization ignored seasonal reproductive characteristics and population sizes. There was no significant (p<0.05) correlation between the modele [file pone.0064970.s003.zip › FileS1/S5.tif]

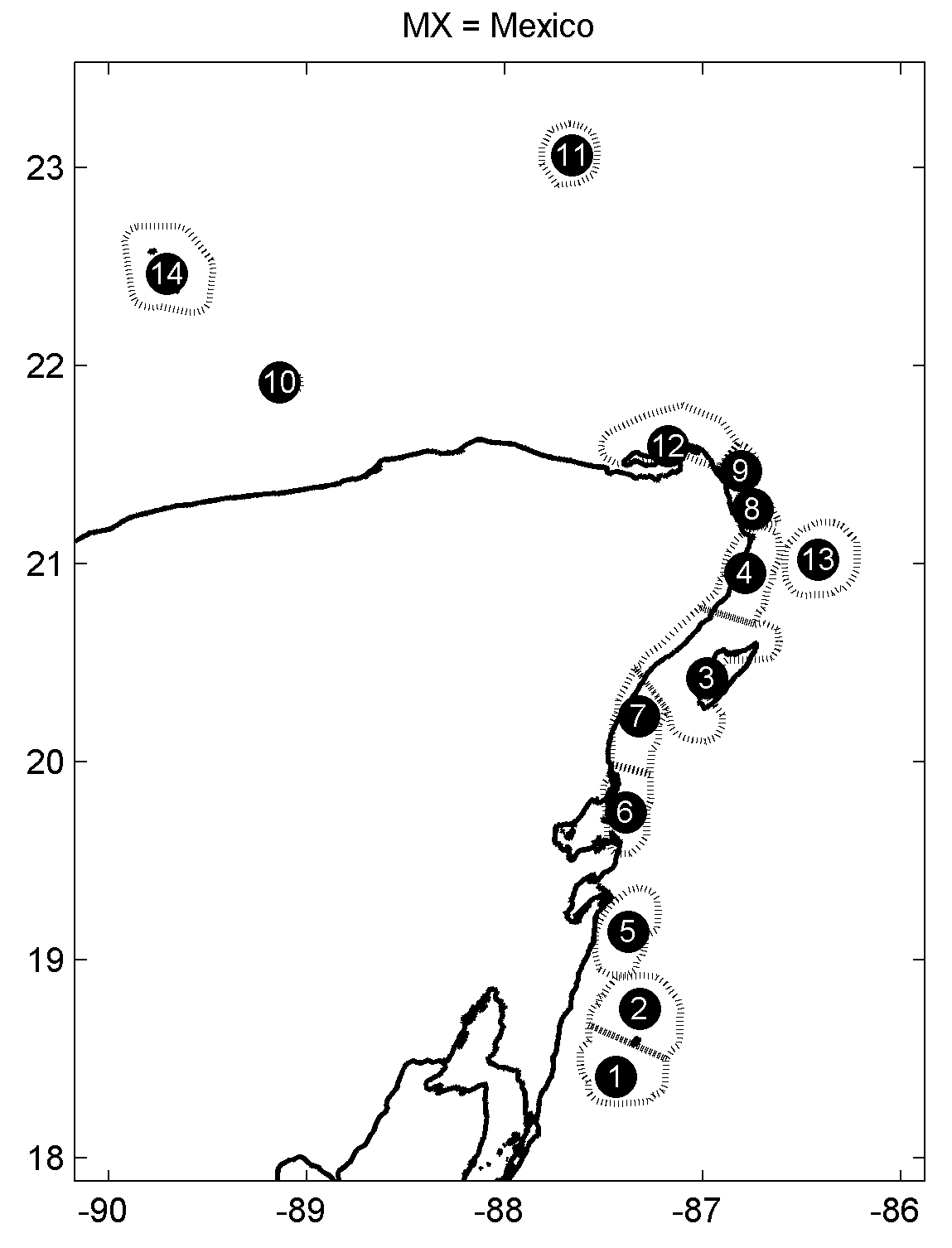

Supplement: File S1 — Contains Figures S1 to S22.: Figure S1 to S20 in File S1. Habitat maps used for the simulation. For each country the habitat sites are shown. Sites within each country are numbered according to the location on the previous figures (2 and 3) reading from left to right along the X axis. All axes are latitude and longitude. BA = Bahamas; CU = Cuba; NI = Nicaragua; FL = Florida; DR = Dominican Republic; MX = Mexico; HO = Honduras; HA = Haiti; BE = Belize; VE = Venezuela; JA = Jamaica; TC = Turks and Caicos; CO = Columbia; PA = Panama; CR = Costa Rica; CA = Cayman Islands; PR = Puerto Rico; LW = Leeward Islands (10 countries); WW = Windward Islands (9 countries); ABC = Aruba, Bonaire, and Curacao. Figure S21 in File S1. A comparison of possible larval sources to the Mexican Quintana Roo coast between two Lagrangian individual based models. The origins of larvae that arrived to habitat on the Quintana Roo coast during April, May, September, and October. Results from Briones-Fourzan are averages between their figures 10 and 11 [29]. Results from our study, using a simulation with passive larvae released equally in magnitude and timing from around the Caribbean (red), and another incorporating vertical migration behavior and larvae released based on reproductive biology (blue). Figure S22 in File S1. The seasonal pattern of observed postlarval arrival compared to modeled predictions, without considering population structure. A comparison of the actual coastal arrival of P. argus postlarvae (red) as compared to modeled predictions (black) over four years at four different locations (Mexico: Bahia de Ascension, Puerto Morales; Florida: Long Key, Big Munson). The Mexican observations are averages from Briones-Fourzan [29]. The Florida FWC observations [35] are of average postlarval arrivals per collector from 2004–2008. The model parameterization ignored seasonal reproductive characteristics and population sizes. There was no significant (p<0.05) correlation between the modele [file pone.0064970.s003.zip › FileS1/S6.tif]

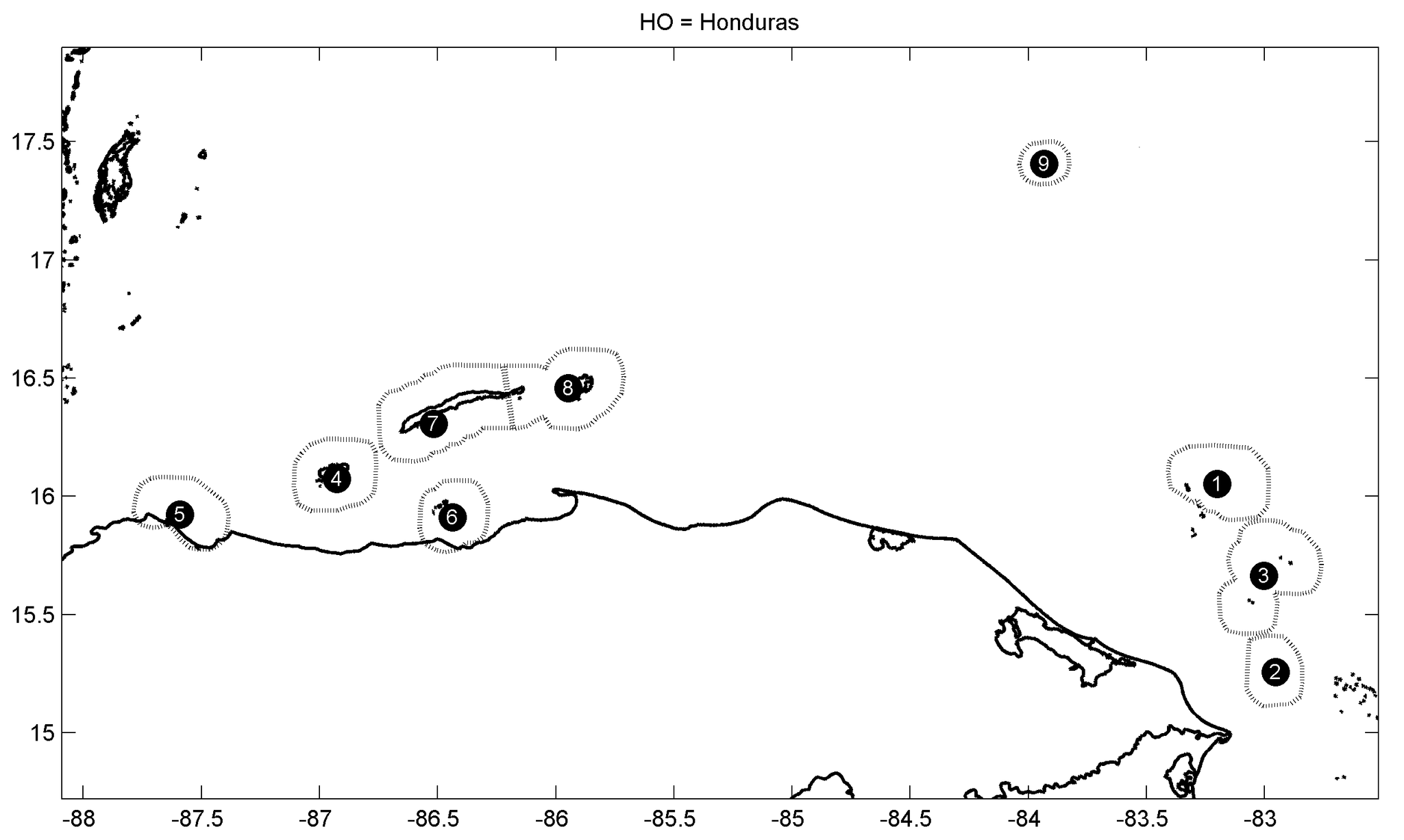

Supplement: File S1 — Contains Figures S1 to S22.: Figure S1 to S20 in File S1. Habitat maps used for the simulation. For each country the habitat sites are shown. Sites within each country are numbered according to the location on the previous figures (2 and 3) reading from left to right along the X axis. All axes are latitude and longitude. BA = Bahamas; CU = Cuba; NI = Nicaragua; FL = Florida; DR = Dominican Republic; MX = Mexico; HO = Honduras; HA = Haiti; BE = Belize; VE = Venezuela; JA = Jamaica; TC = Turks and Caicos; CO = Columbia; PA = Panama; CR = Costa Rica; CA = Cayman Islands; PR = Puerto Rico; LW = Leeward Islands (10 countries); WW = Windward Islands (9 countries); ABC = Aruba, Bonaire, and Curacao. Figure S21 in File S1. A comparison of possible larval sources to the Mexican Quintana Roo coast between two Lagrangian individual based models. The origins of larvae that arrived to habitat on the Quintana Roo coast during April, May, September, and October. Results from Briones-Fourzan are averages between their figures 10 and 11 [29]. Results from our study, using a simulation with passive larvae released equally in magnitude and timing from around the Caribbean (red), and another incorporating vertical migration behavior and larvae released based on reproductive biology (blue). Figure S22 in File S1. The seasonal pattern of observed postlarval arrival compared to modeled predictions, without considering population structure. A comparison of the actual coastal arrival of P. argus postlarvae (red) as compared to modeled predictions (black) over four years at four different locations (Mexico: Bahia de Ascension, Puerto Morales; Florida: Long Key, Big Munson). The Mexican observations are averages from Briones-Fourzan [29]. The Florida FWC observations [35] are of average postlarval arrivals per collector from 2004–2008. The model parameterization ignored seasonal reproductive characteristics and population sizes. There was no significant (p<0.05) correlation between the modele [file pone.0064970.s003.zip › FileS1/S7.tif]

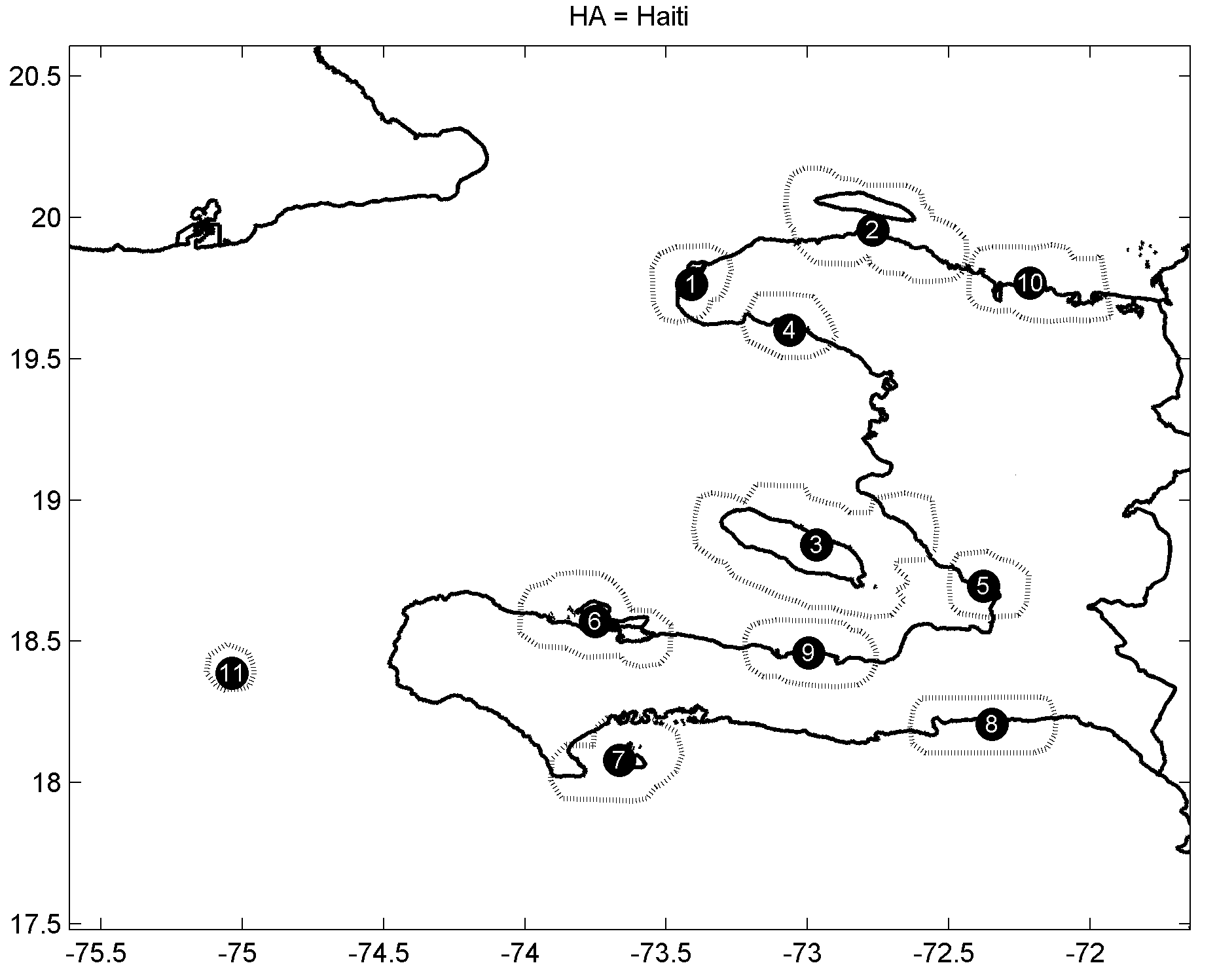

Supplement: File S1 — Contains Figures S1 to S22.: Figure S1 to S20 in File S1. Habitat maps used for the simulation. For each country the habitat sites are shown. Sites within each country are numbered according to the location on the previous figures (2 and 3) reading from left to right along the X axis. All axes are latitude and longitude. BA = Bahamas; CU = Cuba; NI = Nicaragua; FL = Florida; DR = Dominican Republic; MX = Mexico; HO = Honduras; HA = Haiti; BE = Belize; VE = Venezuela; JA = Jamaica; TC = Turks and Caicos; CO = Columbia; PA = Panama; CR = Costa Rica; CA = Cayman Islands; PR = Puerto Rico; LW = Leeward Islands (10 countries); WW = Windward Islands (9 countries); ABC = Aruba, Bonaire, and Curacao. Figure S21 in File S1. A comparison of possible larval sources to the Mexican Quintana Roo coast between two Lagrangian individual based models. The origins of larvae that arrived to habitat on the Quintana Roo coast during April, May, September, and October. Results from Briones-Fourzan are averages between their figures 10 and 11 [29]. Results from our study, using a simulation with passive larvae released equally in magnitude and timing from around the Caribbean (red), and another incorporating vertical migration behavior and larvae released based on reproductive biology (blue). Figure S22 in File S1. The seasonal pattern of observed postlarval arrival compared to modeled predictions, without considering population structure. A comparison of the actual coastal arrival of P. argus postlarvae (red) as compared to modeled predictions (black) over four years at four different locations (Mexico: Bahia de Ascension, Puerto Morales; Florida: Long Key, Big Munson). The Mexican observations are averages from Briones-Fourzan [29]. The Florida FWC observations [35] are of average postlarval arrivals per collector from 2004–2008. The model parameterization ignored seasonal reproductive characteristics and population sizes. There was no significant (p<0.05) correlation between the modele [file pone.0064970.s003.zip › FileS1/S8.tif]

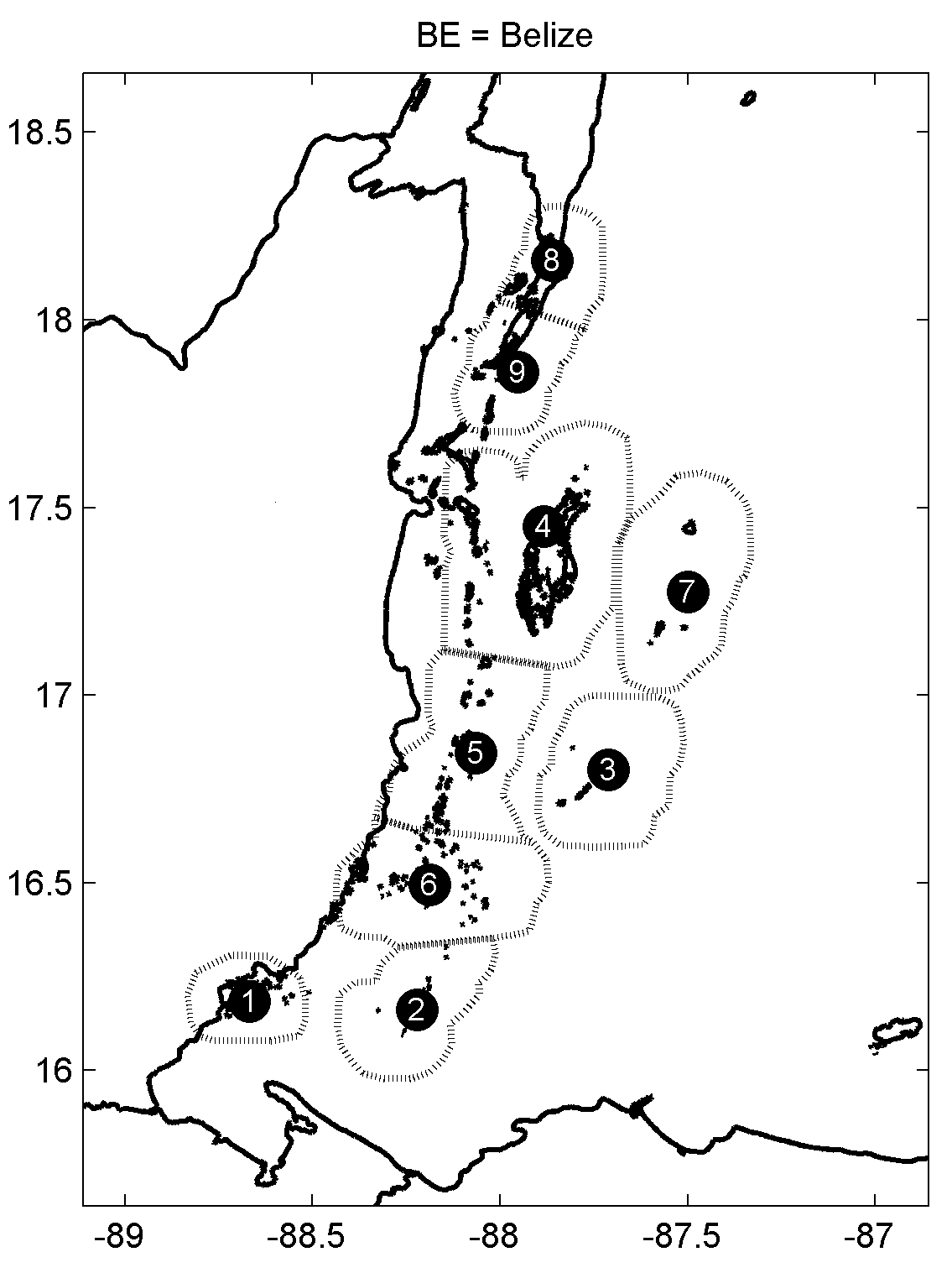

Supplement: File S1 — Contains Figures S1 to S22.: Figure S1 to S20 in File S1. Habitat maps used for the simulation. For each country the habitat sites are shown. Sites within each country are numbered according to the location on the previous figures (2 and 3) reading from left to right along the X axis. All axes are latitude and longitude. BA = Bahamas; CU = Cuba; NI = Nicaragua; FL = Florida; DR = Dominican Republic; MX = Mexico; HO = Honduras; HA = Haiti; BE = Belize; VE = Venezuela; JA = Jamaica; TC = Turks and Caicos; CO = Columbia; PA = Panama; CR = Costa Rica; CA = Cayman Islands; PR = Puerto Rico; LW = Leeward Islands (10 countries); WW = Windward Islands (9 countries); ABC = Aruba, Bonaire, and Curacao. Figure S21 in File S1. A comparison of possible larval sources to the Mexican Quintana Roo coast between two Lagrangian individual based models. The origins of larvae that arrived to habitat on the Quintana Roo coast during April, May, September, and October. Results from Briones-Fourzan are averages between their figures 10 and 11 [29]. Results from our study, using a simulation with passive larvae released equally in magnitude and timing from around the Caribbean (red), and another incorporating vertical migration behavior and larvae released based on reproductive biology (blue). Figure S22 in File S1. The seasonal pattern of observed postlarval arrival compared to modeled predictions, without considering population structure. A comparison of the actual coastal arrival of P. argus postlarvae (red) as compared to modeled predictions (black) over four years at four different locations (Mexico: Bahia de Ascension, Puerto Morales; Florida: Long Key, Big Munson). The Mexican observations are averages from Briones-Fourzan [29]. The Florida FWC observations [35] are of average postlarval arrivals per collector from 2004–2008. The model parameterization ignored seasonal reproductive characteristics and population sizes. There was no significant (p<0.05) correlation between the modele [file pone.0064970.s003.zip › FileS1/S9.tif]
